# EBV epigenetically suppresses the B cell-to-plasma cell differentiation pathway while establishing long-term latency

**DOI:** 10.1371/journal.pbio.2001992

**Published:** 2017-08-03

**Authors:** Christine T. Styles, Quentin Bazot, Gillian A. Parker, Robert E. White, Kostas Paschos, Martin J. Allday

**Affiliations:** Molecular Virology, Department of Medicine, Imperial College London, London, United Kingdom; University of Wisconsin-Madison, United States of America

## Abstract

Mature human B cells infected by Epstein-Barr virus (EBV) become activated, grow, and proliferate. If the cells are infected ex vivo, they are transformed into continuously proliferating lymphoblastoid cell lines (LCLs) that carry EBV DNA as extra-chromosomal episomes, express 9 latency-associated EBV proteins, and phenotypically resemble antigen-activated B-blasts. In vivo similar B-blasts can differentiate to become memory B cells (MBC), in which EBV persistence is established. Three related latency-associated viral proteins EBNA3A, EBNA3B, and EBNA3C are transcription factors that regulate a multitude of cellular genes. EBNA3B is not necessary to establish LCLs, but EBNA3A and EBNA3C are required to sustain proliferation, in part, by repressing the expression of tumour suppressor genes. Here we show, using EBV-recombinants in which both EBNA3A and EBNA3C can be conditionally inactivated or using virus completely lacking the EBNA3 gene locus, that—after a phase of rapid proliferation—infected primary B cells express elevated levels of factors associated with plasma cell (PC) differentiation. These include the cyclin-dependent kinase inhibitor (CDKI) p18^INK4c^, the master transcriptional regulator of PC differentiation B lymphocyte-induced maturation protein-1 (BLIMP-1), and the cell surface antigens CD38 and CD138/Syndecan-1. Chromatin immunoprecipitation sequencing (ChIP-seq) and chromatin immunoprecipitation quantitative PCR (ChIP-qPCR) indicate that in LCLs inhibition of *CDKN2C* (p18^INK4c^) and *PRDM1* (BLIMP-1) transcription results from direct binding of EBNA3A and EBNA3C to regulatory elements at these loci, producing stable reprogramming. Consistent with the binding of EBNA3A and/or EBNA3C leading to irreversible epigenetic changes, cells become committed to a B-blast fate <12 days post-infection and are unable to de-repress p18^INK4c^ or BLIMP-1—in either newly infected cells or conditional LCLs—by inactivating EBNA3A and EBNA3C. In vitro, about 20 days after infection with EBV lacking functional EBNA3A and EBNA3C, cells develop a PC-like phenotype. Together, these data suggest that EBNA3A and EBNA3C have evolved to prevent differentiation to PCs after infection by EBV, thus favouring long-term latency in MBC and asymptomatic persistence.

## Author summary

Epstein-Barr virus (EBV) infection can cause several types of cancer associated with its major target in humans, the mature B cell. Furthermore, EBV is one of the most potent transforming agents ever identified, producing—in vitro—‘immortal’ B lymphoblastoid cell lines (LCLs) with outstanding reliability. However, the near-symbiotic relationship between EBV and its natural host (>95% of human adults are asymptomatically infected) provides a powerful argument that this gamma-herpesvirus did not primarily evolve to be a harmful tumour-causing virus. Consistent with this, we show here that 2 of the potentially oncogenic viral proteins (EBNA3A and EBNA3C) have evolved not to facilitate oncogenic progression but to block plasma cell differentiation in EBV-activated B cells. Specifically, they act to interrupt the gene regulation network that drives activated B cells to become terminally differentiated, quiescent plasma cells, thus allowing for sustained regeneration of virally infected B cells. EBNA3A and EBNA3C achieve this by epigenetically inhibiting expression of cellular genes essential for the differentiation pathway; these include the cyclin-dependent kinase inhibitor p18^INK4c^ and the transcription factor BLIMP-1. This favours the establishment of EBV latency in long-lived memory B cells and therefore helps maintain a ubiquitous, generally asymptomatic infection in human populations.

## Introduction

Epstein-Barr virus (EBV) is a human gamma-herpesvirus that infects about 95% of the world’s adult population. Although infection is nearly always benign, on rare occasions it is etiologically associated with various human cancers, including Burkitt lymphoma (BL), diffuse large B cell lymphoma (DLBCL), Hodgkin’s lymphoma, and post-transplant lymphoproliferative disease (PTLD). EBV is also the primary cause of acute infectious mononucleosis, aka glandular fever [1–3]. Although EBV can be found in epithelial, natural killer (NK), and T cells, it preferentially infects mature B cells. Infection of primary human CD19^+ve^ B lymphocytes ex vivo results in their activation, growth, and sustained proliferation so that lymphoblastoid cell lines (LCLs) are established. These cells have a B-blast–like phenotype, carry EBV genomes as extra-chromosomal episomes, and express a limited number of viral latency-associated factors; these include 6 nuclear proteins (EBNA1, EBNA2, EBNA3A, EBNA3B, EBNA3C, and EBNA-LP), 2 membrane-associated proteins (LMP1 and LMP2), 2 small non-coding RNAs, and several microRNAs (reviewed in [2,4]). This pattern of viral gene expression is known as the latency III or growth program. Infection of mature B cells in vivo is thought to initiate a similar program of activation and growth, but only transient proliferation, because under the influence of CD4^+ve^ T helper (Th) lymphocytes these B cells differentiate in the lymphoid structures called germinal centres (GC) to form a pool of memory B cells (MBC) carrying latent EBV [5–8]. These EBV genome–containing, long-lived MBC can then enter the peripheral circulation and, expressing no viral proteins, form a major site of lifelong EBV persistence. To complete the life cycle, reactivation of EBV into a program of gene expression that leads to replication and release of virions is thought to occur in a small number of B cells differentiating into antibody-secreting plasma cells (PCs) [9].

The EBNA3 latency-associated proteins are a family of 3 large (approximately 160kD) EBV nuclear antigens expressed from 3 genes arranged in tandem within a complex transcription unit only functional in B cells (reviewed in [10]). All 3 proteins can act as transcription factors that regulate host gene expression [11]. They do not bind DNA directly, but, via contacts with cellular cofactors, are largely targeted to chromatin at gene promoters and/or distal regulatory elements [12–15]. Often, regulation of transcription involves long-distance chromatin interactions (chromosome ‘looping’) between promoter and enhancer elements mediated by EBNA3 proteins [16–19].

EBNA3A and EBNA3C have been widely reported to reduce expression of various cell cycle regulatory factors found in latently infected B cells. For instance, EBNA3A and EBNA3C together repress expression of the pro-apoptotic BCL2-family member BIM and the senescence-inducing cyclin-dependent kinase inhibitor (CDKI) p16^INK4a^—2 tumour suppressors that would otherwise contribute to an oncogenic stress response resulting from virus infection driving unscheduled DNA synthesis and cell proliferation (reviewed in [20]). In addition, EBNA3A and EBNA3C directly repress transcription of the p16^INK4a^-related CDKI, p15^INK4b^ [21] and—by inducing precursors of oncogenic microRNAs miR-221 and miR-222—inhibit expression of CIP/KIP family CDKIs, p57^KIP2^ and p27^KIP1^ [19]. Furthermore, EBNA3A- and EBNA3C-mediated repression of transcription of genes encoding the related INK4 CDKIs p16^INK4a^ (*CDKN2A*) and p15^INK4b^ (*CDKN2B*) is associated with binding of these viral factors to chromatin in and around the gene loci and subsequent deposition of repressive histone marks by polycomb protein complexes [21]. By targeting these various genes, operationally EBNA3A and EBNA3C can behave as oncoproteins, but these same functions facilitate early MYC-driven cell proliferation soon after infection [10]. In contrast, EBNA3B is not necessary to establish or maintain LCL proliferation [10] and can act as a tumour suppressor in a humanized-mouse model and in some human tumours—at least in part—by facilitating immune cell trafficking and T cell surveillance [22].

Of all the mammalian CDKIs, there is one specifically linked to B cell function: the INK4 family member p18^INK4c^, encoded by the *CDKN2C* gene. Although biochemically and structurally p18^INK4c^ very closely resembles p16^INK4a^, functionally it appears to be specialized for differentiation [23,24].

The requirement for p18^INK4c^ in PC differentiation was first indicated by a significant reduction of antibody secretion in p18^INK4c^-deficient mice during primary and secondary immune responses. It was confirmed when ectopic p18^INK4c^ expression was shown to rescue PC differentiation in vitro [24,25]. Consistent with these observations, reduced proliferation and concurrent differentiation towards a PC phenotype correlates with increased p18^INK4c^ expression in mitogen-activated CD19^+ve^ human B cells [26]. Moreover, increased amounts of p18^INK4c^ (relative to its preferred target, the cyclin-dependent kinase CDK6) are linked to differentiation in other cell lineages, including osteoclasts and myeloid cells [23].

There is general agreement that in vivo, differentiation of B cells to PCs can be initiated from various cell populations, including antigen-activated extra-follicular B cells (e.g., marginal B cells and B1 cells), from GC B cells, and from circulating MBC [27–29]. In each case the genome-wide transcription patterns of the B cells and derived PCs are significantly different, indicating that there is substantial reprogramming of gene expression networks as differentiation proceeds [28]. This is a very complex process involving many factors, but there is an emerging consensus of opinion that at least 3 transcriptional regulators are essential for differentiation to occur efficiently (reviewed in [28,30–32]). These factors are B lymphocyte-induced maturation protein-1 (BLIMP-1), interferon-regulatory factor 4 (IRF4), and a specifically spliced form of X-box binding protein-1 (XBP-1). Although EBV-transformed LCLs generally express high levels of IRF4 [33,34], they express little BLIMP-1 or XBP-1 (see below). It is the lack of sufficient BLIMP-1 that probably explains why EBV-activated B cells generally remain self-renewing blasts and fail to undergo default terminal differentiation to antibody-secreting PCs unless additional endogenous or paracrine signals are applied.

Until now, the relationship between EBV and the B cell differentiation specialized CDKI p18^INK4c^ has not been reported. Considering EBNA3A and EBNA3C are well-established transcriptional regulators of CDKI, it was therefore of particular interest to investigate whether they also regulate p18^INK4c^ and subsequently to determine whether EBNA3A and EBNA3C play a specific role in the regulation of B cell-to-PC differentiation—a role directly attributed to p18^INK4c^.

Here, using novel EBV recombinants encoding conditional versions of both EBNA3A and EBNA3C, we show that when these latent proteins are non-functional, mature human B cells infected ex vivo show elevated expression of 2 key regulators of PC differentiation, p18^INK4c^ and BLIMP-1. Chromatin immunoprecipitation sequencing (ChIP-seq) and chromatin immunoprecipitation quantitative PCR (ChIP-qPCR) data are consistent with EBNA3A and EBNA3C binding to regulatory elements and directly epigenetically repressing the *CDKN2C* (p18^INK4c^) and *PRDM1* (BLIMP-1) genes. Furthermore, cell surface marker analysis indicates a PC–like phenotype when EBNA3A and EBNA3C are inactive. We suggest a major physiological function of EBNA3A and EBNA3C is to block terminal differentiation to PCs, the default pathway in B cells normally activated by antigens without help from T cells.

## Results

### EBNA3 proteins prevent activation of p18^INK4c^ transcription after EBV infection

**Fig 1:**
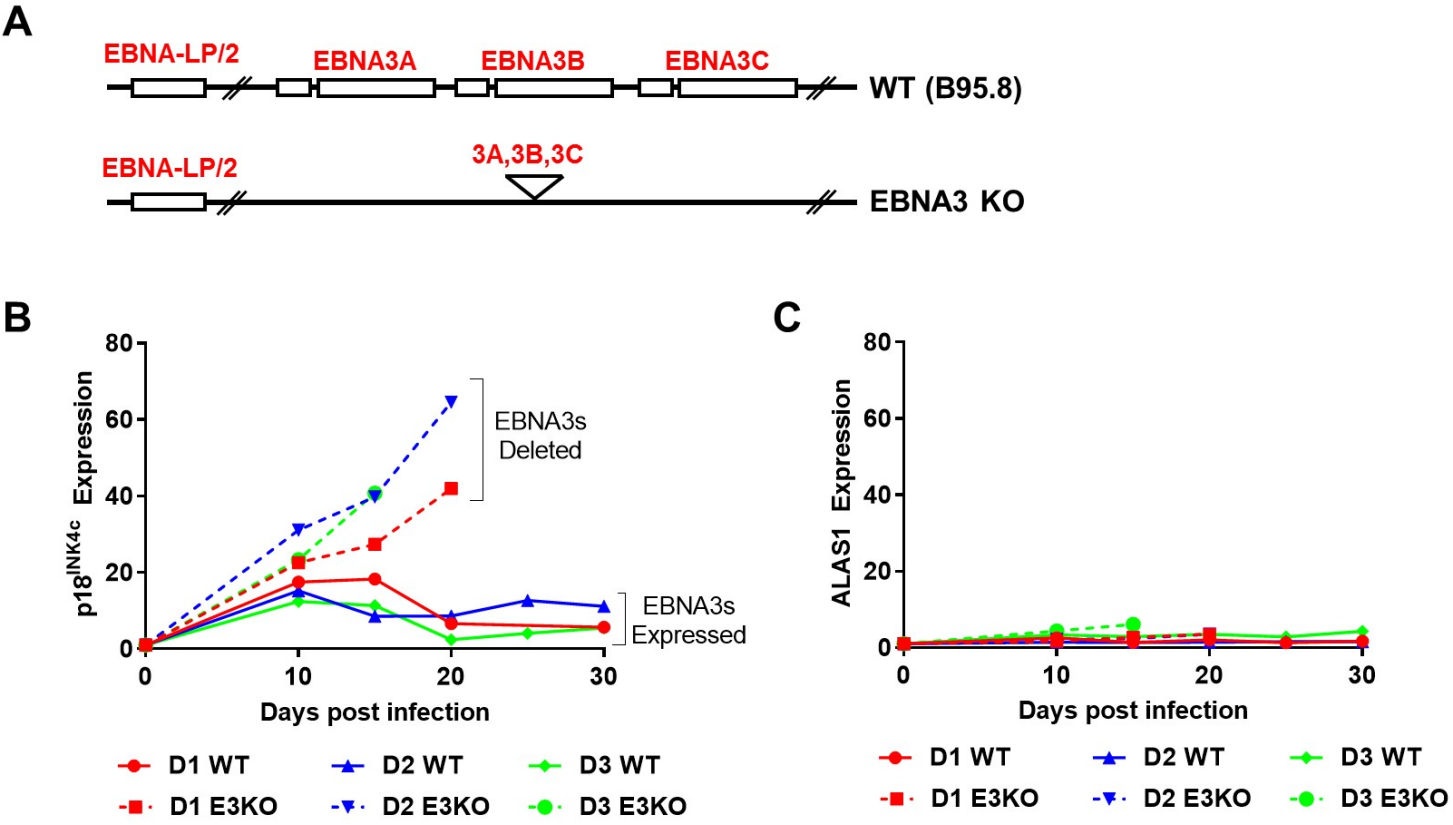
Deletion of the EBNA3 locus results in de-repression of p18^INK4c^ transcription after Epstein-Barr virus (EBV) infection. Schematic (aligned, but not to scale) of partial EBV B95.8-BAC genome and a recombinant from which the EBNA3A/3B/3C locus has been deleted (EBNA3KO; [11]) (**A**). CD19^+ve^ purified B cells from 3 donors (D1, D2, D3) were infected with EBNA3KO or ‘WT’ (B95.8-BAC) recombinant EBV and cultured for 30 days. RNA samples were taken at the times indicated after infection and quantitative PCR (qPCR) analysis performed on each. *CDKN2C* (p18^INK4c^, **B**) and the housekeeping gene *ALAS1* (**C**) relative mRNA expression was normalised to the endogenous control *GNB2L1* and fold changes are shown relative to uninfected B cells at day 0. Error bars show the standard deviation of qPCR triplicates for each sample. Analysis of EBNA3KO-infected cells at later time points was not always possible because of large amounts of cell death in the culture. Numerical data for this figure can be found at osf.io/97zrj.

To assess the involvement of the EBNA3 proteins in regulation of p18^INK4c^ we infected CD19^+ve^ peripheral B cells with an EBV recombinant from which the whole EBNA3 locus was deleted (EBNA3KO) (Fig 1A; [11]). We saw reproducibly—using B cells from independent donors—that 15–20 days post-infection (pi) there was a substantial increase in the expression of p18^INK4c^, compared to cells infected with the prototypical lab strain (referred to hereafter as ‘WT’ B95.8-BAC), where all EBNA3 proteins are expressed (Fig 1B). Relative expression of the control housekeeping gene ALAS1 was unaffected by the absence of the EBNA3 proteins (Fig 1C). With substantial increases in p18^INK4c^ expression seen in all donors when the entire EBNA3 locus is deleted, it is likely an EBNA3 protein, or a combination of EBNA3 proteins, is responsible for repression of p18^INK4c^ expression.

### Construction and validation of the double-conditional EBNA3A-ERT2 and EBNA3C-ERT2 EBV-recombinant (3A3CERT2)

**Fig 2:**
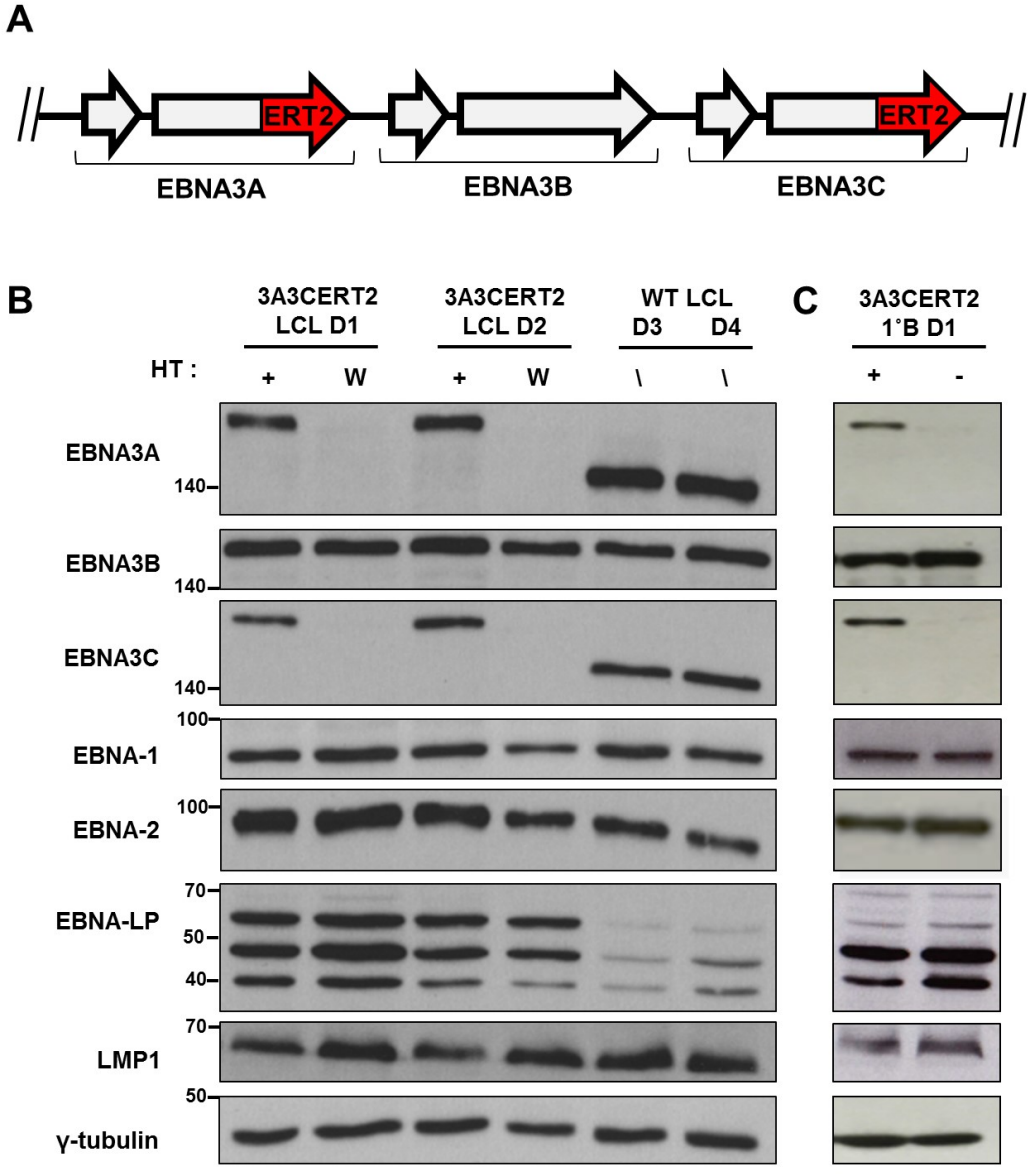
Validation of double-conditional 3A3CERT2-infected cells. (**A**) Schematic showing ERT2 fusion to the C-terminus of EBNA3A and EBNA3C open reading frames (3A3CERT2). (**B**) Expression of Epstein-Barr virus latency-associated proteins EBNA1, EBNA2, EBNA3A, EBNA3B, EBNA3C, EBNA-LP, and LMP1 was demonstrated by western blotting extracts from 3A3CERT2 lymphoblastoid cell lines (LCLs) derived from 2 donors (3A3CERT2 LCL D1, 3A3CERT2 LCL D2) cultured with 4-hydroxytamoxifen (HT) (+) or washed and grown without HT for 30 days (W), ‘WT’ (B95.8-BAC) LCLs derived from 2 donors (WT D3, WT D4) and (**C**) Primary CD19^+ve^ B cells 20 days post-infection with 3A3CERT2 recombinant (1°B D1) cultured with (+) or without (-) HT. In each blot γ-tubulin was used as a loading control; molecular weight markers are shown in kDa.

As indicated above, the currently available data suggest that EBNA3B is unlikely to be involved in the regulation of p18^INK4c^ or in the B cell transformation/immortalization process, with EBNA3A and EBNA3C widely considered the principle EBV-mediated regulators of CDKI [11,22]. Therefore, to focus on the relative contributions of EBNA3A and EBNA3C, and to formally exclude EBNA3B playing a role in the context of the triple EBNA3KO EBV, we constructed a recombinant virus in which EBNA3A and EBNA3C could be co-regulated, while EBNA3B remained constitutively active (see schematic in Fig 2A). The 3A3CERT2 virus—that expresses functional EBNA3A and EBNA3C only in the presence of the activating ligand (4-hydroxytamoxifen [HT]) of the modified-oestrogen receptor—was constructed using bacterial artificial chromosome (BAC) technology and its genomic structure was confirmed (see Materials and methods). LCLs were established using CD19^+ve^ B cells from 2 independent ‘buffy coats’ from anonymous donors (3A3CERT2 LCL D1 and 3A3CERT2 LCL D2) infected with 3A3CERT2 and cultured in the continuous presence of HT. When HT was removed, with the cells washed in fresh medium and re-cultured, the EBNA3 fusions were sequestered in the cytoplasm [35–37]. Western blot analyses of these cells showed that the stability of the EBNA3A- and EBNA3C-ERT2 fusion proteins was dependent on HT in the growth-medium (Fig 2B). EBNA3A- and EBNA3C-ERT2 proteins have an increased molecular weight due to the ERT2-fusion (Fig 2B).

Expression of other EBV latency-associated proteins—including EBNA3B—was unaffected by this switch in EBNA3A/EBNA3C functionality. The expression of latency-associated proteins in 3A3CERT2 LCLs is comparable with those in ‘WT’ B95.8 LCLs with the exception of EBNA-LP. However, EBNA-LP levels have been shown to be donor dependent [19,38], and although levels of this protein are lower in ‘WT’ B95.8 LCLs, they are comparable between 3A3CERT2 LCLs grown with or without HT.

Consistent with the results from these established LCLs, examining freshly infected primary B cells 20 days pi, grown either with or without HT, revealed similar patterns of latent protein expression (see Fig 2C for example). This confirms our recombinant 3A3CERT2 virus has simultaneous conditional expression of both EBNA3A and EBNA3C, while maintaining expression of other latency-associated proteins.

### EBNA3A and EBNA3C are required to prevent activation of *CDKN2C*, the gene encoding p18^INK4c^

**Fig 3:**
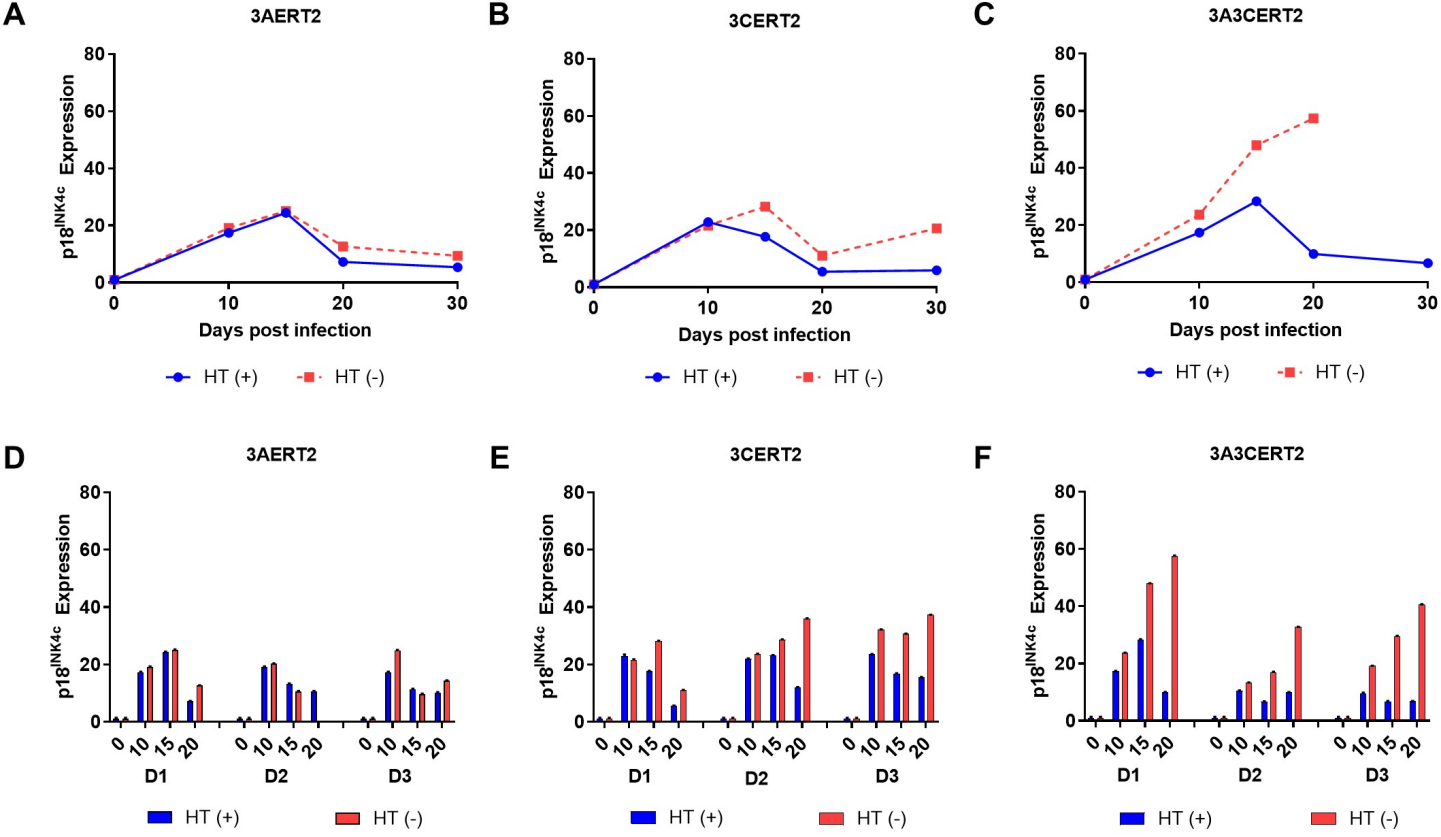
EBNA3A and EBNA3C are required to prevent activation of *CDKN2C* (p18^INK4c^). CD19^+ve^ purified B cells were infected with 3AERT2 (**A** and **D**), 3CERT2 (**B** and **E**), or 3A3CERT2 (**C** and **F**) recombinant Epstein-Barr virus and cultured for 30 days with (+) or without (-) 4-hydroxytamoxifen (HT). RNA samples were taken at the times after infection indicated and quantitative PCR (qPCR) analysis performed. *CDNK2C* (p18^INK4c^) relative mRNA expression was normalised to the endogenous control *GNB2L1* and fold change is shown relative to uninfected B cells at day 0. Error bars show the standard deviation of qPCR triplicates for each sample. Analysis of HT (-) infected cells at later time points was not always possible because of large amounts of cell death in the culture. Similar analysis from 2 additional donors (D2, D3) is also shown for comparison (**D, E, F**). Numerical data for this figure can be found at osf.io/97zrj.

In order to establish whether EBNA3A and/or EBNA3C regulate the expression of the p18^INK4c^-encoding gene *CDKN2C* in newly infected B cells, an experiment was performed using cells from a single donor infected with EBV recombinants conditional for EBNA3A (3AERT2), EBNA3C (3CERT2), or both proteins (3A3CERT2). Cells were cultured with or without HT, from the day of infection for up to 30 days. Samples of cells were harvested at the time of infection and after 10, 15, 20, and 30 days and mRNA extracted for analysis by reverse transcription quantitative PCR (RT-qPCR). There was little difference in the level of p18^INK4c^ mRNA whether EBNA3A was functional (3AERT2 +HT) or inactive (3AERT2 -HT) (Fig 3A). Similar analysis of 3CERT2-infected cells suggested that non-functional EBNA3C (-HT) results in a small but sustained increase in p18^INK4c^ mRNA (Fig 3B). However, by far the most striking increase in p18^INK4c^ mRNA was seen when both EBNA3A and EBNA3C were inactive after infection (3A3CERT2 -HT) (Fig 3C). These data were consistent in magnitude and kinetics with experiments using the triple knockout virus (Fig 1B). The results were consistent and reproducible in infections of B cells derived from 3 independent donors (Fig 3D–3F). We conclude that inactivating EBNA3A alone has very little effect on p18^INK4c^ expression, and inactivating EBNA3C alone has a modest but reproducible effect. However, inactivating EBNA3A and EBNA3C simultaneously leads to a very robust activation of p18^INK4c^ expression during the first 3 weeks after infection, confirming EBNA3A/EBNA3C-mediated regulation of *CDKN2C*. Analysis of control housekeeping genes (e.g., *ALAS1*) showed no change in the mRNA level (S1A–S1C Fig), but similar analysis of p16^INK4a^ mRNA confirmed what has been widely reported previously [38–40]; that is, inactivating EBNA3C or, to a lesser extent, EBNA3A leads to increased p16^INK4a^ expression. Inactivating both EBNA3A and EBNA3C together produced an additive increase in p16^INK4a^ mRNA (see S1D–S1F Fig). Analysis of p15^INK4b^ mRNA from the 20 days pi samples showed that inactivation of both EBNA3A and EBNA3C induced a substantial increase in p15^INK4b^ expression (S2A Fig), indicating that *CDKN2A* and *CDKN2B* are co-regulated.

### EBNA3A and EBNA3C are required to prevent activation of *PRDM1*, the gene encoding BLIMP-1

**Fig 4:**
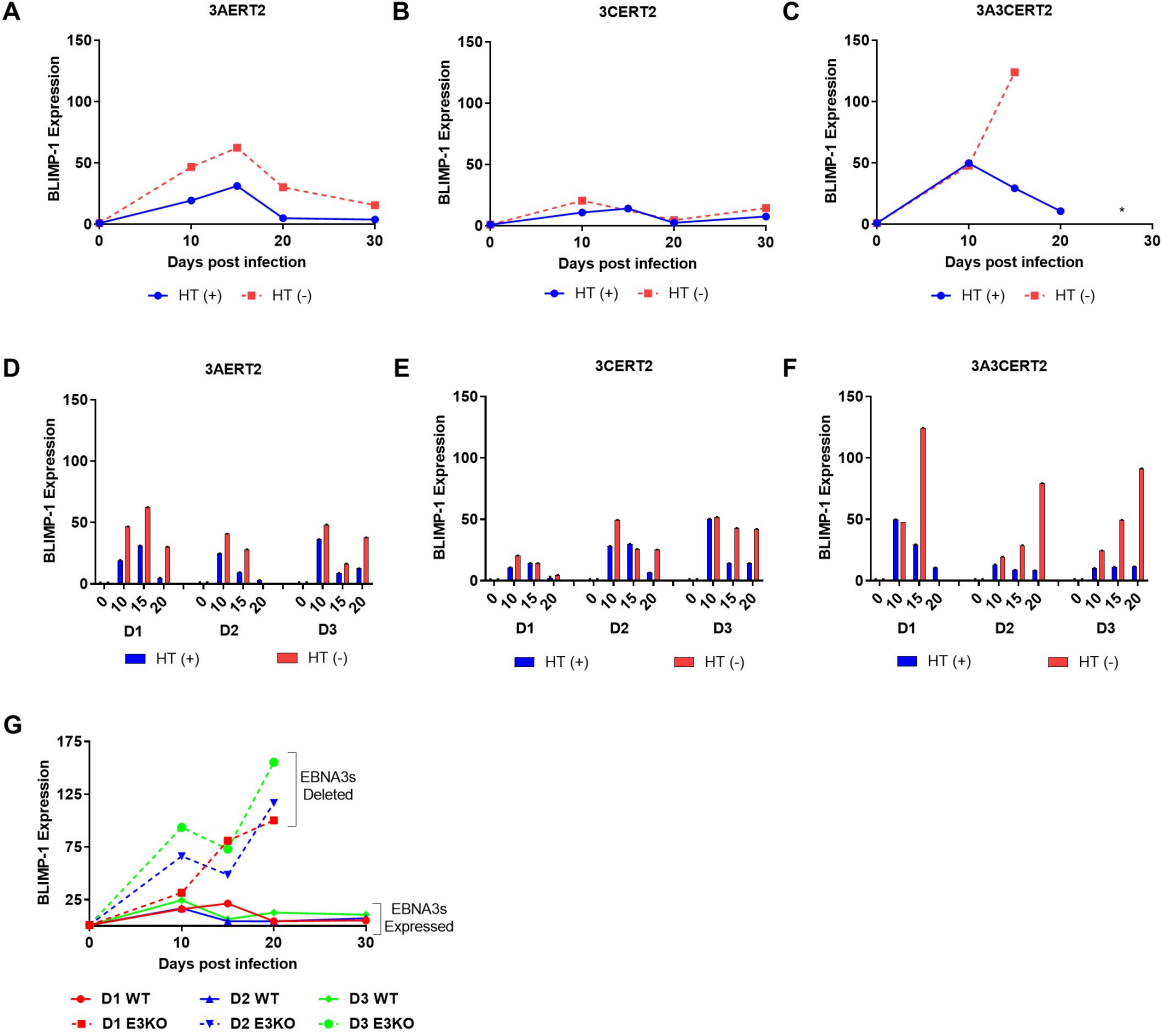
EBNA3A and EBNA3C are required to prevent activation of *PRDM1* (B lymphocyte-induced maturation protein-1 [BLIMP-1]). CD19^+ve^ purified B cells were infected with 3AERT2 (**A** and **D**), 3CERT2 (**B** and **E**), or 3A3CERT2 (**C** and **F**) recombinant Epstein-Barr virus (EBV) and cultured for 30 days with (+) or without (-) 4-hydroxytamoxifen (HT). RNA samples were taken at the times after infection indicated and quantitative PCR (qPCR) analysis performed. *PRDM1* (BLIMP-1) relative mRNA expression was normalised to the endogenous control *GNB2L1* and fold change is shown relative to uninfected B cells at day 0. Error bars show the standard deviation of qPCR triplicates for each sample. Analysis of HT (-) infected cells at later time points was not always possible because of large amounts of cell death in the culture. Similar analysis from 2 additional donors (D2, D3) is also shown for comparison (**D, E, F**). (**G**) CD19^+ve^ purified B cells from 3 donors (D1, D2, and D3) were infected with EBNA3KO or ‘WT’ (B95.8-BAC) recombinant EBV, RNA samples were taken and qPCR analysis performed as for (**A–F**). Numerical data for this figure can be found at osf.io/97zrj.

Because inactivation of EBNA3A and EBNA3C produced a marked increase in the expression of p18^INK4c^ mRNA after infection with EBV, and this CDKI has been definitively linked to PC differentiation (see Introduction), we asked whether BLIMP-1, the key transcriptional regulator of this pathway, was also a target of EBV. RNAs from the cells used in the experiments shown in Fig 3, were subjected to RT-qPCR using primers specific for BLIMP-1 mRNA. This showed BLIMP-1 is regulated in a very similar way to p18^INK4c^ (Fig 4). In particular, BLIMP-1 mRNA expression after infection with recombinants in which both EBNA3A and EBNA3C were inactivated (3A3CERT2 -HT) were greatly increased (Fig 4C and 4F), similar to p18^INK4c^ mRNA (compare Figs 3F and 4F) and comparable to the results using EBNA3KO virus (Fig 4G). Inactivation of EBNA3A (3AERT2 -HT) had a very modest but reproducible effect (Fig 4A and 4D), whereas inactivation of EBNA3C alone (3CERT2 -HT) had very little effect on BLIMP-1 expression (Fig 4B and 4E).

We conclude that EBV, via the transcription factors EBNA3A and EBNA3C, regulates at least 2 critical cellular genes involved in PC differentiation: p18^INK4c^ and BLIMP-1.

### Repression of p18^INK4c^ and BLIMP-1 initiated by EBNA3A and EBNA3C becomes irreversible by day 12pi

**Fig 5:**
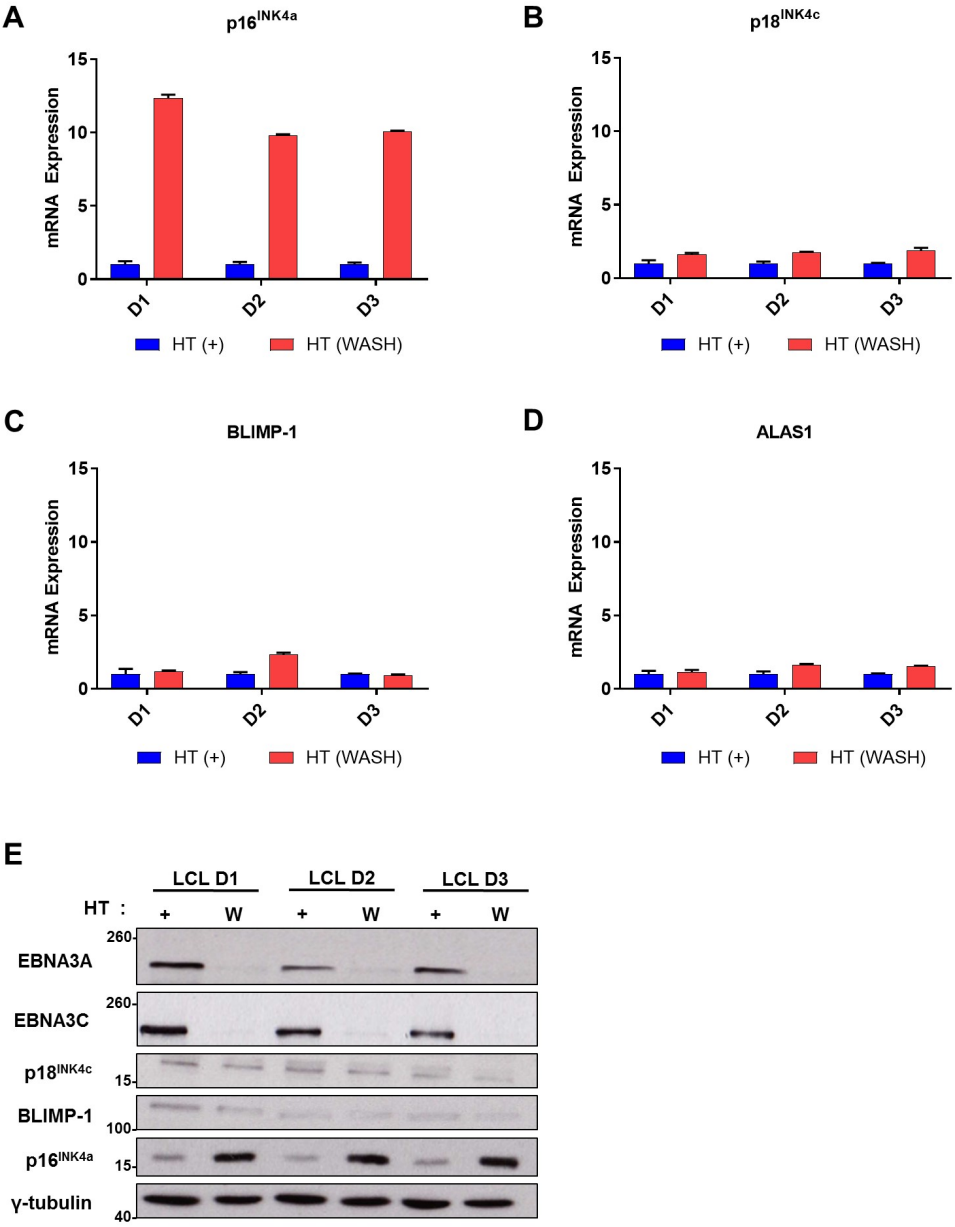
Repression of p18^INK4c^ and B lymphocyte-induced maturation protein-1 (BLIMP-1) transcription is not reversible by inactivating EBNA3A/EBNA3C in lymphoblastoid cell lines (LCLs). Established conditional 3A3CERT2 LCLs from 3 donors (D1, D2, D3) were cultured with (+) or washed and grown without (WASH) 4-hydroxytamoxifen (HT) for 30 days. Analysis of expression of (**A**) *CDKN2A* (p16^INK4a^), (**B**) *CDKN2C* (p18^INK4c^), (**C**) *PRDM1* (BLIMP-1), and (**D**) *ALAS1* (control housekeeping gene) mRNA was performed by quantitative PCR (qPCR) and relative mRNA expression was normalised to the endogenous control *GNB2L1*, with fold change shown relative to LCLs grown with (+) HT. Error bars indicate the standard deviation of qPCR triplicates for each sample. (**E**) Western blotting extracts of the same cells show expression of EBNA3A, EBNA3C, p18^INK4c^, BLIMP-1, and p16^INK4a^; γ-tubulin was used as a loading control; molecular weight markers are shown in kDa. Numerical data for this figure can be found at osf.io/97zrj.

The genes encoding p16^INK4a^ and p15^INK4b^ are epigenetically repressed in established LCLs [10,38]. However, if conditional EBNA3C is inactivated by the removal of the activating ligand HT from the growth medium, the repressed state of the *CDKN2A* locus is reversed and the levels of p16^INK4a^ RNA and protein increase (Fig 5A and 5E; [38,39]). The transcription of various other genes regulated by EBNA3C (e.g., *COBLL1* and *AICDA*; [12,17]) or EBNA3A and EBNA3C (e.g., *BIM/BCL2L11*; [41]) are also reversibly regulated. In order to determine whether p18^INK4c^ and BLIMP-1 are regulated by EBNA3A and EBNA3C in a similar way, LCLs derived from donors D1, D2, and D3 cells were investigated. LCLs established with 3A3CERT2 virus were continuously cultured in HT (+HT) for >60 days, then half the cells were cultured with no HT (HT WASH). Unlike the expression of p16^INK4a^ and p15^INK4b^ mRNA that was de-repressed within 30 days without HT (Fig 5A and S2B Fig), similar analyses consistently showed no increase in p18^INK4c^ or BLIMP-1 mRNA (Fig 5B and 5C), suggesting repression of these 2 differentiation-associated loci by EBNA3A and EBNA3C is not reversible under similar conditions. As expected, expression of the control housekeeping gene *ALAS1* was unaffected by the inactivation of EBNA3A and EBNA3C (Fig 5D). Analysis by western blotting confirmed the same trend at a protein level (Fig 5E).

**Fig 6:**
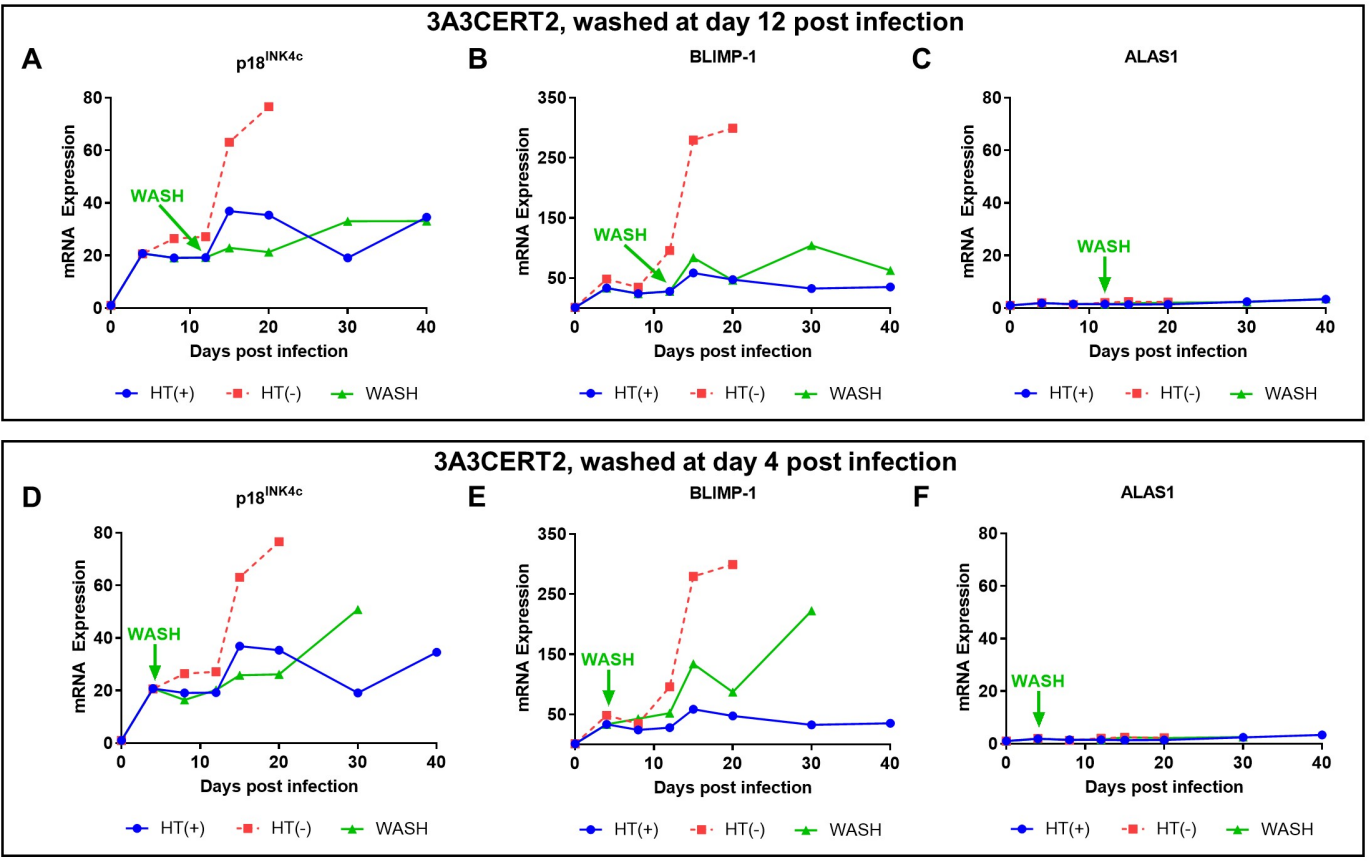
Transcription of p18^INK4c^ and B lymphocyte-induced maturation protein-1 (BLIMP-1) is repressed early after infection with Epstein-Barr virus (EBV). CD19^+ve^ purified B cells from 1 independent donor were infected with 3A3CERT2 recombinant EBV and cultured for 30 days with 4-hydroxytamoxifen (HT) (+), without HT (-), or HT was removed after 12 or 4 days (WASH) as indicated. RNA samples were taken at the times after infection indicated and quantitative PCR (qPCR) analysis performed. *CDKN2C* (p18^INK4c^; **A** and **D**), *PRDM1* (BLIMP-1; **B** and **E**), and the control housekeeping gene *ALAS1* (**C** and **F**) relative mRNA expression was normalised to the endogenous control *GNB2L1* with fold change shown relative to uninfected B cells at day 0. Error bars show the standard deviation of qPCR triplicates for each sample. Analysis of HT (-) and day 4 washed infected cells at later time points was not possible because of large amounts of cell death in the culture. Numerical data for this figure can be found at osf.io/97zrj.

To determine how soon this repression becomes irreversible, CD19^+ve^ primary B cells from an independent donor were infected with the 3A3CERT2 recombinant EBV and the activating ligand (HT) was added at the time of infection. HT was then washed out from the medium after 4 or 12 days. By 12 days pi, repression of both p18^INK4c^ and BLIMP-1 can no longer be reversed—that is, cells exposed to active EBNA3A/EBNA3C for 12 days appear to be committed to self-renewal rather than differentiation (Fig 6A–6C; a second independent donor is shown in S3 Fig). If HT is removed from the population 4 days after infection there can be partial activation of p18^INK4c^ and BLIMP-1, suggesting that EBNA3A and EBNA3C act earlier in some cells (Fig 6D–6F and S3D–S3F Fig). Taken together, these data demonstrate EBV commits to EBNA3A and EBNA3C mediated irreversible repression of p18^INK4c^ and BLIMP-1 within 12 days of primary B cell infection.

### Chromatin immunoprecipitations reveal binding of both EBNA3A and EBNA3C to *CDKN2C* and *PRDM1* loci

**Fig 7:**
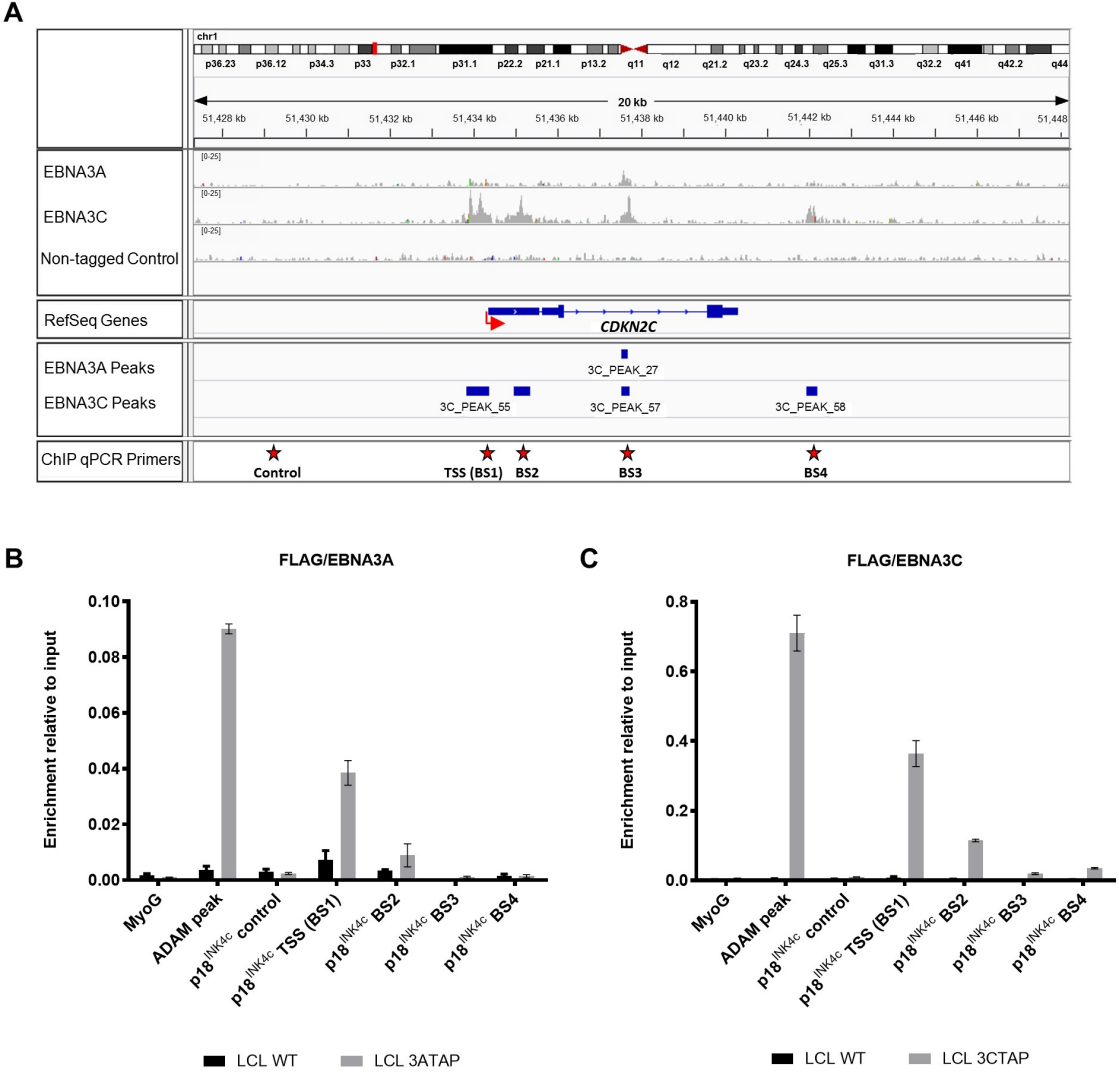
EBNA3A and EBNA3C bind to the *CDKN2C* locus. (**A**) Aligned reads for EBNA3A, EBNA3C, and the negative control (non-tagged lymphoblastoid cell line [LCL]) at *CDKN2C* locus, visualized using Integrated Genomics Viewer (IGV). The red arrow shows the transcription start site (TSS) of *CDKN2C*. Red stars indicate EBNA3A and EBNA3C binding site primers (BS1, BS2, BS3, and BS4) as well as control region primers used for chromatin immunoprecipitation quantitative PCR (ChIP-qPCR). (**B**) ChIP-qPCR analyses using anti-Flag antibody to precipitate 3A-TAP and chromatin associated with it in LCL 3A-TAP was performed. As a control for antibody specificity, similar ChIP was performed using LCL infected with WT (B95.8-BAC; LCL WT). Primers for the *myoglobin* promoter (MyoG) were used for qPCR as a negative control, whereas primers for known EBNA3A/3C binding sites at the *ADAM28/ADAMDEC1* intergenic enhancer (ADAM) were used as positive controls of EBNA3 binding. Values represent ratio of chromatin precipitated, after correction for IgG, relative to 2.5% of input. Standard deviations are calculated from qPCR triplicates for each sample. The ChIP shown here is 1 representative example from at least 3 independent experiments. (**C**) As in (**B**) but using LCL 3C-TAP in order to precipitate 3C-TAP protein and associated chromatin. Numerical data for this figure can be found at osf.io/97zrj.

**Fig 8:**
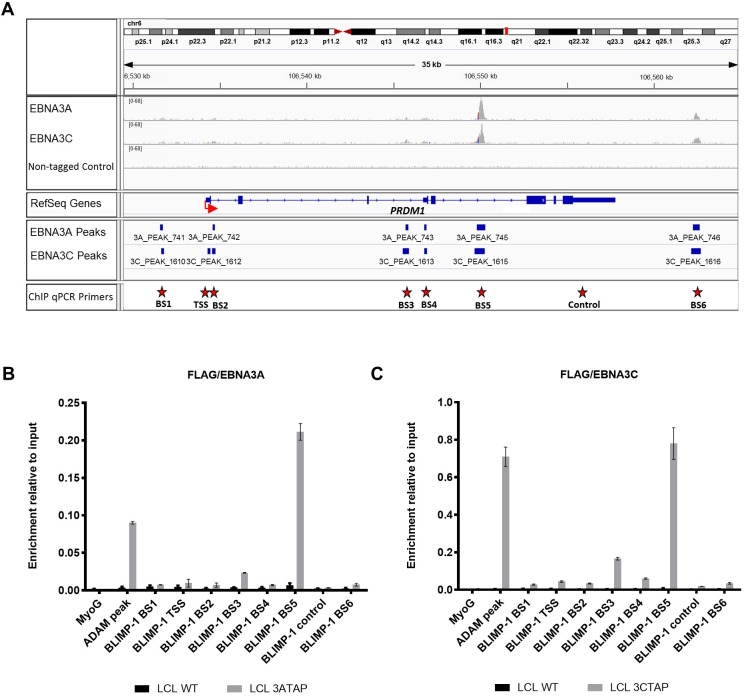
EBNA3A and EBNA3C bind to the *BLIMP-1/PRDM1* locus. (**A**) Aligned reads for EBNA3A, EBNA3C, and the negative control (non-tagged lymphoblastoid cell line [LCL]) at *PRDM1* locus, visualized using Integrated Genomics Viewer (IGV). The red arrow shows the transcription start site (TSS) of *PRDM1*. Red stars show the positions of EBNA3A and EBNA3C binding site primers (BS1, BS2, BS3, BS4, BS5, and BS6) as well as TSS primers and control region primers used for chromatin immunoprecipitation quantitative PCR (ChIP-qPCR). (**B**) ChIP-qPCR analyses using anti-Flag antibody to precipitate 3A-TAP and chromatin associated with it in LCL 3A-TAP was performed. As a control for antibody specificity, similar ChIP was performed using LCL infected with WT (B95.8-BAC; LCL WT). Primers for the *myoglobin* promoter (MyoG) were used for qPCR as negative control, whereas primers for known EBNA3A/3C binding sites at the *ADAM28/ADAMDEC1* intergenic enhancer (ADAM) were used as positive controls of EBNA3 binding. Values represent ratio of chromatin precipitated, after correction for IgG, relative to 2.5% of input. Standard deviations are calculated from qPCR triplicates for each sample. The ChIP shown here is 1 representative example from at least 3 independent experiments. (**C**) As in (**B**) but using LCL 3C-TAP in order to precipitate 3C-TAP protein and associated chromatin. Numerical data for this figure can be found at osf.io/97zrj.

Interrogating a recent ChIP-seq analysis performed on LCLs established with EBV-recombinants expressing epitope-tagged EBNA3A or EBNA3C revealed multiple binding peaks on chromatin for both EBNA3A and EBNA3C distributed across both the *CDKN2C* (p18^INK4c^) and *PRDM1* (BLIMP-1) loci (Fig 7 and Fig 8; [15]). At the *CDKN2C* locus (Fig 7A) both the raw sequence reads and peaks identified by the high stringency model-based analysis of ChIP-seq (MACS) algorithm revealed 4 substantial EBNA3C binding sites (BS1-4), of which BS1 spans the predicted transcription start site (TSS; -490 to +44) for p18^INK4c^ mRNA. EBNA3C appears to bind BS1 and BS2 very efficiently, but considerable EBNA3C is also found at a discrete intronic site, BS3. Overall, the distribution of EBNA3A appears similar, but the binding is probably slightly less efficient—with only 1 MACS peak being called, at BS3. To verify EBNA3A and EBNA3C occupancy there, binding of tagged EBNA3A (EBNA3A-TAP) and tagged EBNA3C (EBNA3C-TAP) at these loci were determined in LCLs by ChIP-qPCR. These data (Fig 7B and 7C) confirm that BS1 and BS2 (both located proximal to the p18^INK4c^ TSS) are the predominant binding sites.

The ChIP-seq raw sequence reads [15] for the *PRDM1* locus show that of the 6 peaks (BS1-6) identified by MACS algorithm binding both EBNA3A and EBNA3C, the intronic peak (BS5) is most prominent (Fig 8A). ChIP-qPCR confirmed that BS5 is by far the most efficient binding site for both EBNA3A and EBNA3C (Fig 8B and 8C). We therefore conclude that EBNA3A and EBNA3C are targeted to chromatin within the genes encoding p18^INK4c^ and BLIMP-1, indicating a direct repressive role.

### Sustained EBV-mediated repression of p18^INK4c^ and BLIMP-1 expression involves PRC2 and the repressive histone modification H3K27me3

**Fig 9:**
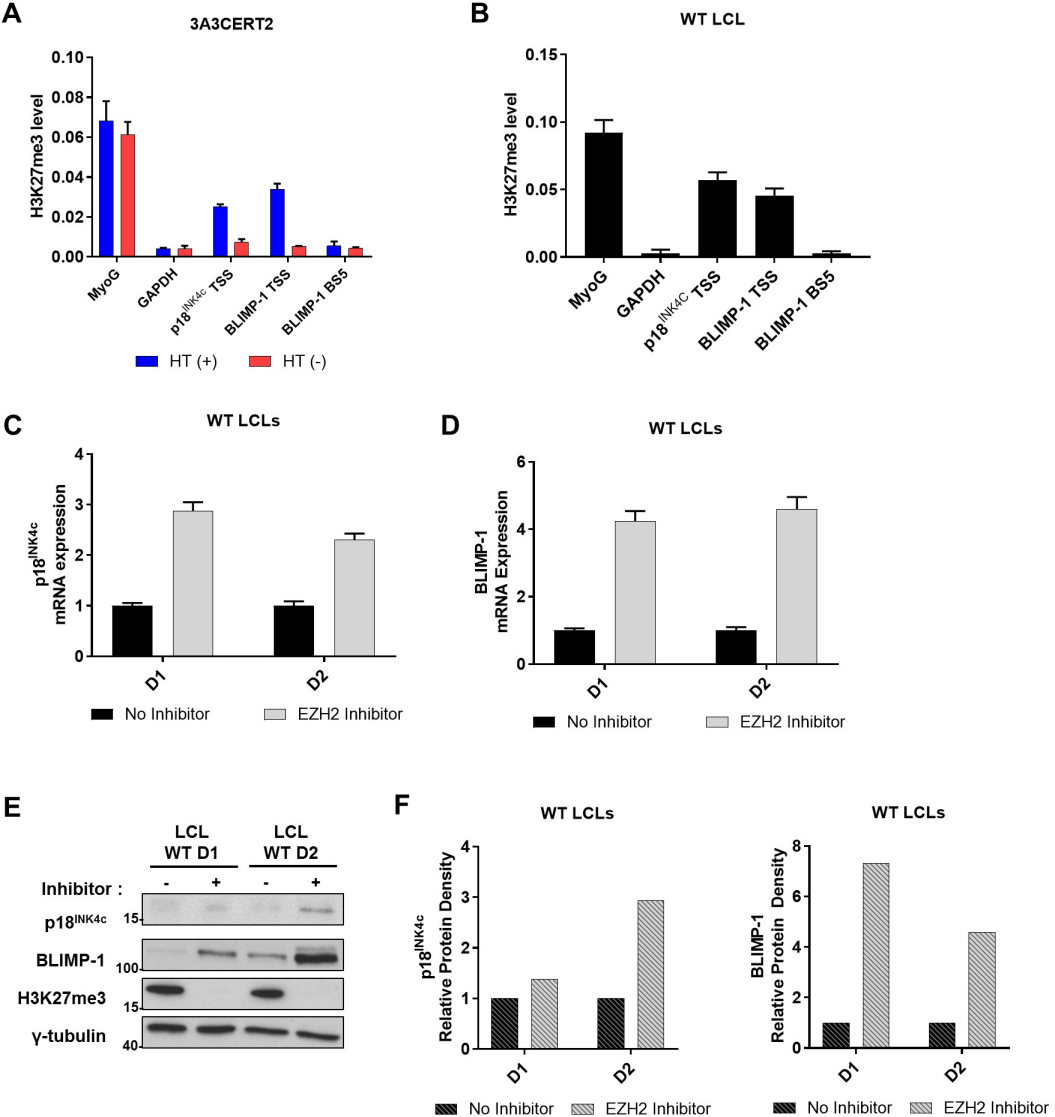
Polycomb repressor complex 2 (PRC2) and the histone modification H3K27me3 are associated with repression of *CDKN2C* and *PRDM1* by EBNA3A and EBNA3C. Repressive chromatin modifications were analysed by chromatin immunoprecipitation (ChIP) assays using antibody specific for the tri-methylated form of lysine 27 on histone H3 (H3K27me3). Enrichment at B lymphocyte-induced maturation protein-1 (BLIMP-1) transcription start site (TSS), the main BLIMP-1 EBNA3 binding site (BS5), p18^INK4c^ TSS, and *GAPDH* were assessed. Values represent ratio of chromatin precipitated, after correction for IgG, relative to 2.5% of input. Standard deviations are calculated from quantitative PCR (qPCR) triplicates for each sample. The ChIPs shown here are 1 representative example from at least 3 independent experiments. Cells were harvested from CD19^+ve^ purified B cells infected with 3A3CERT2 recombinant EBV and cultured for 20 days with (+) or without (-) 4-hydroxytamoxifen (HT) (**A**) and an established lymphoblastoid cell line (LCL) (**B**). Established ‘WT’ (B95.8-BAC) LCLs from 2 different donors (LCL WT D1 and LCL WT D2) were treated with the Enhancer of Zeste Homologue-2 (EZH2) inhibitor GSK126 for 20 days. Analysis of expression of *CDKN2C* (p18^INK4c^, **C**) and *PRDM1* (BLIMP-1, **D**) was performed by qPCR and mRNA expression was normalized to the endogenous control *GNB2L1*. Standard deviations are calculated from qPCR triplicates for each sample. (**E**) Western blotting extracts of the same cells as **C**/**D** show expression of p18^INK4c^, BLIMP-1, and total H3K27me3. In each case γ-tubulin was used as a loading control, and molecular weight markers are shown in kDa. (**F**) p18^INK4c^ and BLIMP-1 western blot protein bands were analysed by ImageJ software and represented as bar diagrams based on internal loading control γ-tubulin. Numerical data for this figure can be found at osf.io/97zrj.

On the basis of all the data presented above (and previous reports [12,15,38,41]), we assumed that EBNA3A and EBNA3C, by binding to the chromatin in the *CDKN2C* and *PRDM1* genes, would produce changes in histone modifications on local chromatin. At several EBNA3A/EBNA3C target genes, repression of transcription has been shown to correlate with the repressive histone mark H3K27me3 that is normally catalysed by the Enhancer of Zeste Homologue-2 (EZH2), an essential component of the polycomb repressor complex 2 (PRC2) [41,42]. In order to determine whether the levels of H3K27me3 correlate with the repression of *CDKN2C* and *PRDM1* by EBNA3A and EBNA3C, 3A3CERT2-infected CD19^+ve^ B cells (grown with or without HT) were harvested 20 days pi and subjected to further ChIP analysis. When EBNA3A and EBNA3C are active (3A3CERT2 +HT), H3K27me3 occupancy is increased across both genes—in particular at sites proximal to each TSS (Fig 9A). Moreover, analysis of an established LCL confirmed a significant steady-state distribution of H3K27me3 when EBNA3A and EBNA3C are active (Fig 9B).

In order to confirm that the histone mark H3K27me3, and therefore PRC2, is not only present but also involved in p18^INK4c^ and BLIMP-1 repression in LCLs, we made use of the small molecule GSK126, a specific inhibitor of EZH2 [43]. Two independent LCLs (LCL WT D1 and LCL WT D2) were treated with the inhibitor for 20 days and harvested for RNA and protein extraction. The level of p18^INK4c^ (Fig 9C) and BLIMP-1 mRNA (Fig 9D) increased in both LCLs after EZH2 inhibition. Analysis of protein levels by western blot also confirmed the de-repression of BLIMP-1, and to a lesser extent p18^INK4c^, and that the GSK126 treatment effectively abrogated the total level of H3K27me3 in LCL (Fig 9E). As expected, proliferation in GSK126-treated cells is reduced, with EBNA3A and EBNA3C protein expression modestly increased (S4 Fig).

Taken together, we conclude that PRC2 and the repressive histone modification H3K27me3 are involved in EBNA3A and EBNA3C mediated repression of p18^INK4c^ and BLIMP-1.

### Differential expression of PC differentiation associated factors in EBNA3A/EBNA3C-null cells

**Fig 10:**
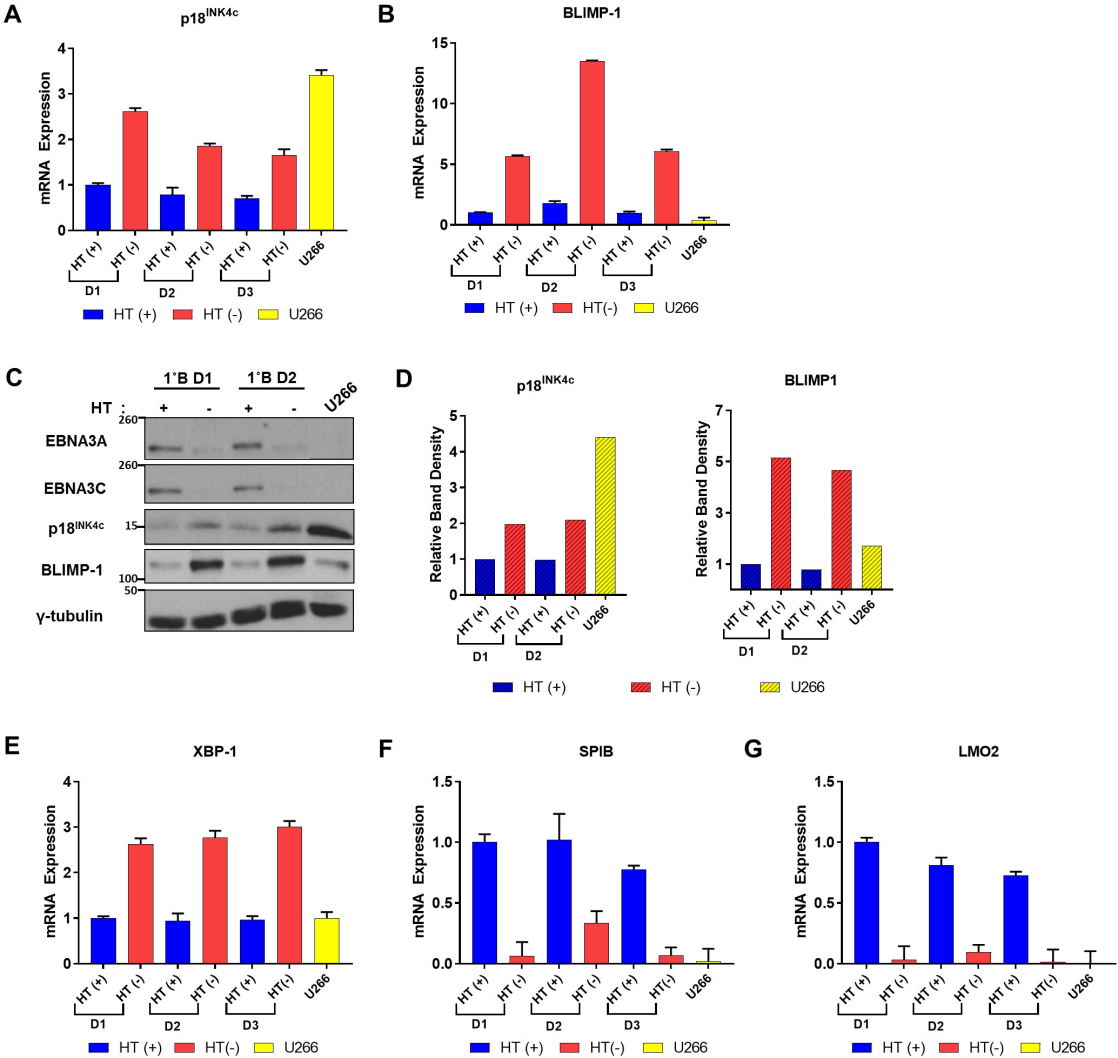
Expression of plasma cell differentiation factors in 3A3CERT2-infected cells and the plasmacytoma-derived cell line U266. RNA samples were taken from U266 cells and CD19^+ve^ purified B cells from 3 independent donors (D1, D2, D3) infected with 3A3CERT2 recombinant Epstein-Barr virus and grown for 20 days with (+) or without (-) 4-hydroxytamoxifen (HT), and quantitative PCR (qPCR) performed. (**A**) *CDKN2C* (p18^INK4c^) and (**B**) *PRDM1* (B lymphocyte-induced maturation protein-1 [BLIMP-1]) mRNA expression was normalized to the endogenous control *GNB2L1* with fold change shown relative to D1 grown with HT (+) 0. Error bars show the standard deviation of qPCR triplicates for each sample. (**C**) Western blotting extracts from the same cells show expression of EBNA3A, EBNA3C, p18^INK4c^, and BLIMP-1; γ-tubulin was used as a loading control; molecular weight markers are shown in kDa. (**D**) p18^INK4c^ and BLIMP-1 and western blot protein bands were analysed by ImageJ software and represented as bar diagrams based on internal loading control γ-tubulin. (**E,F,G**) As in (**A**,**B**) but *XBP-1*, *LMO2*, and *SPIB* mRNA analysed. Numerical data for this figure can be found at osf.io/97zrj.

Considering both p18^INK4c^ and BLIMP-1 are associated with PC differentiation, we wanted to assess whether expression of these factors in EBNA3A/EBNA3C-null cells were physiologically comparable to levels expressed in PCs and therefore sufficient to drive differentiation. As multiple myeloma cell lines are derived from neoplasms arising from terminally differentiated PCs, we utilized the myeloma/plasmacytoma-derived cell line U266 as a PC model [44,45]. We assessed levels of p18^INK4c^ and BLIMP-1 by RT-qPCR and western blotting in U266 alongside 3A3CERT2-infected cells (+/-HT) from multiple donors at 20 days pi (Fig 10).

Levels of p18^INK4c^ mRNA in 3A3CERT2-infected cells with non-functional EBNA3A and EBNA3C are lower but comparable to U266 levels (depending on the donor) and consistently higher than when EBNA3A and EBNA3C are active (Fig 10A). On the other hand, levels of BLIMP-1 in these cells far exceed that seen in U266 across all donors (Fig 10B). Western blotting and quantification by densitometry confirmed these RNA changes in p18^INK4c^ and BLIMP-1 translate to protein, with respective levels consistent between RNA and protein expression in U266 and 3A3CERT2-infected cells (Fig 10C and 10D). BLIMP-1 is a DNA-binding zinc finger transcription factor and many of its target genes have been characterized [30,46,47]. To determine whether increasing BLIMP-1 was functional in our cells and transcriptionally activating target gene *XBP-1*, and repressing the target genes *LMO2* and *SPIB*, we extended our mRNA analysis of 3A3CERT2-infected cells. Consistent with a physiological increase in functional BLIMP-1, and concurrent p18^INK4c^ increase, *XBP-1* transcription consistently increases around 4-fold when EBNA3A and EBNA3C are inactivated, exceeding levels seen in U266, which are comparable to EBV-infected cells with functional EBNA3A and EBNA3C (Fig 10E). Furthermore, transcription of the BLIMP-1–repressed genes *LMO2* and *SPIB* is substantially reduced when EBN3A and EBNA3C are inactivated, reaching levels as low as U266 cells (Fig 10F and 10G), indicating that the activated BLIMP-1 retains its repressive activity. *LMO2* and *SPIB* transcriptional repression and *XBP-1* activation were followed during the first 30 days after infection of primary CD19^+ve^ B cells with the 3A3CERT2 virus +/- HT, and were found to be consistent with BLIMP-1 activation and comparable to the phenotype seen after infection with EBNA3KO virus relative to ‘WT’ (B95.8-BAC) infection (S5 Fig).

Alongside BLIMP-1, the transcription factor IRF4 is also implicated in the B cell-to-PC differentiation pathway (See Introduction, [34]). Western blots showed that newly infected B cells and established LCLs express substantial amounts of IRF4—as much, if not more, than in U266. The level of expression is unaffected by EBNA3A/EBNA3C functionality (S6 Fig).

Therefore, we have found that in cells lacking functional EBNA3A and EBNA3C, factors required for PC differentiation are induced to levels comparable to, or greater than, levels in U266 cells.

### PC surface markers and immunoglobulins production is elevated in EBNA3A/EBNA3C-null cells

The PC–like phenotype of 3A3CERT2 EBV-infected cells with non-functional EBNA3A and EBNA3C was further studied and the presence of PC surface markers was assessed by flow cytometric analysis (FCA). There is widespread agreement that the defining characteristics of PCs are expression of CD138 (aka Syndecan-1) and elevated levels of CD38, alongside expression of CD27 and down-regulation/absence of CD20; therefore, these markers where our focus [48–50].

**Fig 11:**
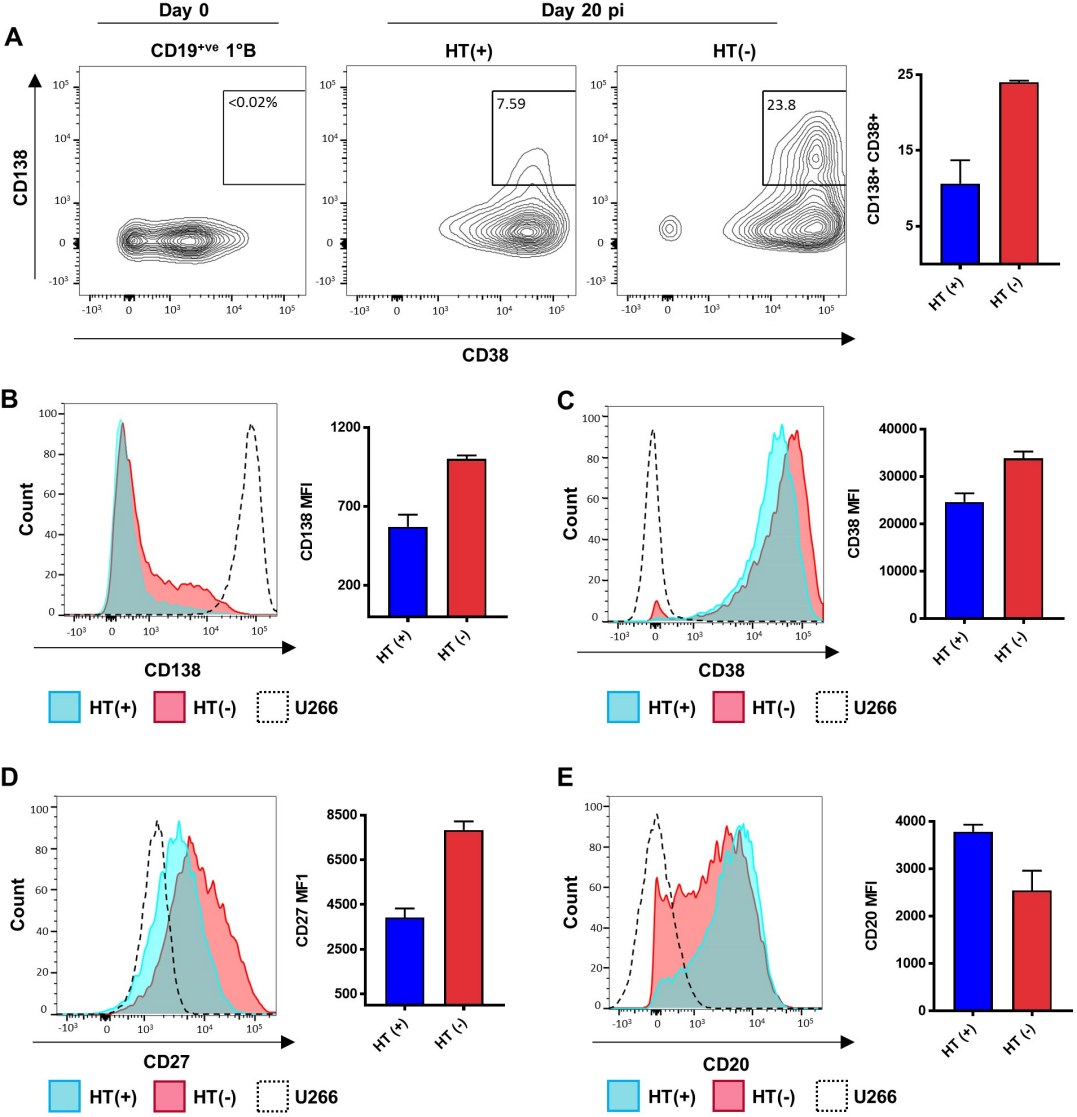
Flow cytometric profiles of EBNA3A/EBNA3C-null cells are consistent with the plasmablast/plasma cell phenotype. U266 cells (black) and CD19^+ve^ purified B cells infected with 3A3CERT2 recombinant Epstein-Barr virus and cultured with (+, blue) or without (-, red) 4-hydroxytamoxifen (HT) for 20 days were analysed for CD138, CD38, CD27, and CD20 expression by flow cytometry. (**A**) Contour plots show CD38 and CD138 surface expression; the quadrant value represents the percentage of live single cells expressing both markers. (**B**) Mean percentage of CD138^+^ and CD38^high^ cells from 2 donors. Histograms show expression of CD138 (**C**), CD38 (**D**), CD27 (**E**), and CD20 (**F**), and corresponding mean fluorescence intensity of positive cells from 2 donors. Error bars show standard deviation from 2 donors. Histograms and contour plots shown are 1 representative example from 2 independent experiments. Numerical data for this figure can be found at osf.io/97zrj. Abbreviation: pi, post-infection.

Approximately 20% of infected cells lacking EBNA3A and EBNA3C express surface markers consistent with a PC–like phenotype at day 20 pi when defined as CD138^+^ and CD38^high^ (Fig 11; a second independent donor is shown in S7 Fig). However, overall there is a notable shift in expression of both CD138 and CD38 in EBNA3A/EBNA3C-null cells, compared to those where the proteins are functional, indicating the majority of cells are responding to the absence of EBNA3A and EBNA3C (Fig 11B and 11C).

We also found a distinct shift in expression in other PC surface markers, with CD27 levels elevated in cells with non-functional EBNA3A and EBNA3C, exceeding those found in our PC control U266, alongside distinct reduction in CD20, approaching levels seen in U266 (Fig 11D and 11E and S7D and S7E Fig).

As cells with non-functional EBNA3A/EBNA3C at 20 days pi appear to express a plasma cell–like phenotype, we utilized FCA to assess whether they were producing immunoglobulin (Ig), a hallmark of PCs. We induced Ig-producing PC in vitro through exposure to CD40-ligand and IL21 (described by [51]) as a second PC model to assess levels of IgG and IgM in EBNA3A/EBNA3C-null cells, as U266 cells do not produce these Igs [44,52]. We confirmed p18^INK4c^ and BLIMP-1 were activated in these cells; however, as with U266, levels were greater in cells with inactive EBNA3A and EBNA3C (S8 Fig).

**Fig 12:**
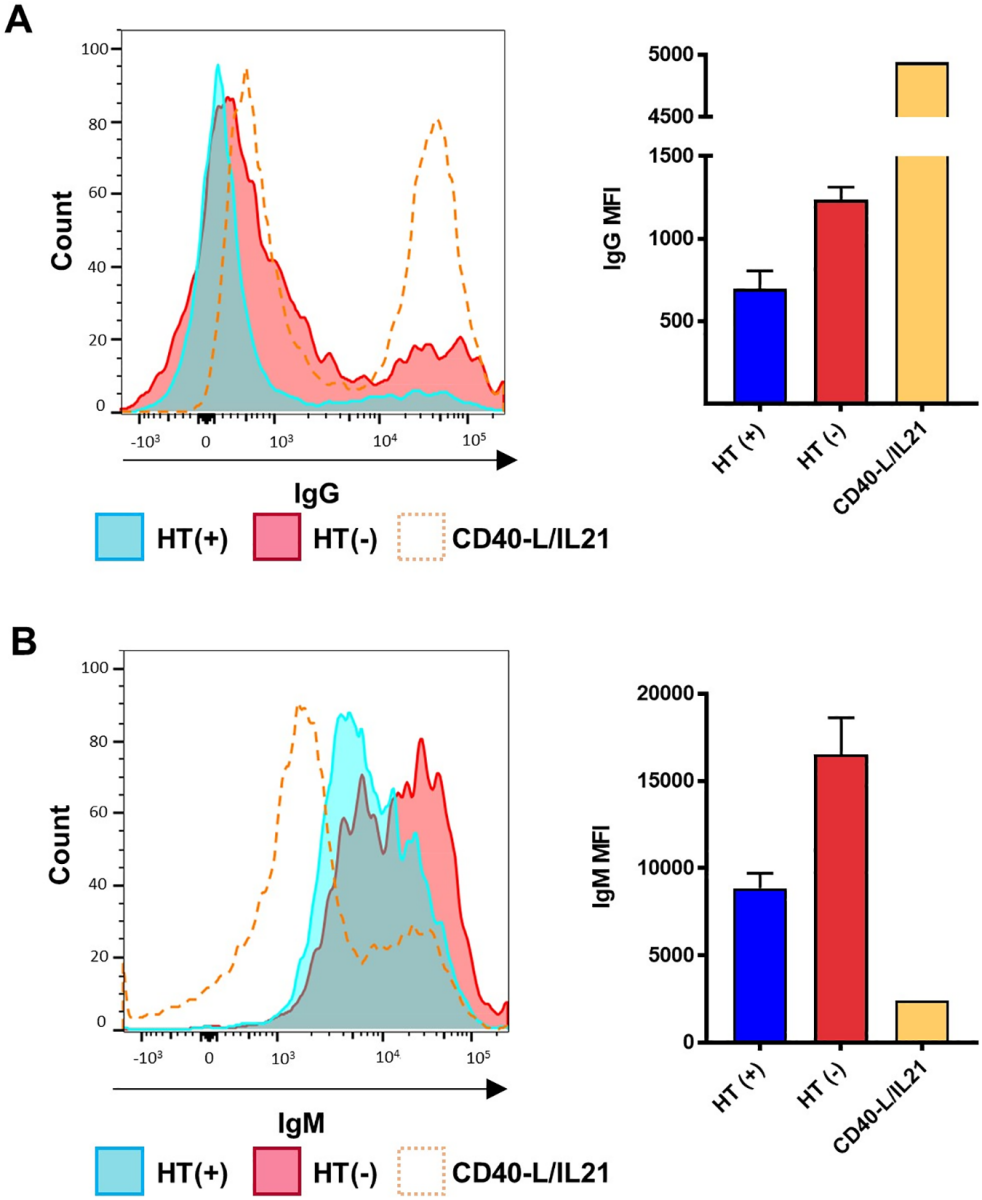
Recombinant Epstein-Barr virus (EBV)-infected cells express IgG and IgM. CD19^+ve^ purified B cells were infected with 3A3CERT2 recombinant EBV and cultured with (+, blue) or without (-, red) 4-hydroxytamoxifen (HT) for 20 days, then analysed for IgG and IgM expression by flow cytometry. Histograms show expression of IgG (**A**) and IgM (**B**) alongside corresponding mean fluorescence intensity of positive cells from 2 donors. Plasma cells induced in vitro with CD40-ligand/IL21 were used as a control (orange). Error bars show standard deviation from 2 donors. Histograms shown are 1 representative example from 2 independent experiments. Numerical data for this figure can be found at osf.io/97zrj.

Using FCA we found a distinct shift in IgG expression in cells lacking EBNA3A/EBNA3C, with a notable population reaching expression levels comparable to induced PCs (Fig 12; a second independent donor is shown in S9 Fig). Furthermore, there is a notable shift in IgM levels with increased expression in all cells lacking EBNA3A and EBNA3C, with overall levels exceeding that of the induced control across both infections but with the high-producing 3A3CERT2 population exhibiting similar levels as the high producers of the CD40-L/IL21–treated cells (Fig 12B and S9B Fig). Additionally, using immunohistochemistry to analyse 3A3CERT2-infected cells (grown with or without HT) at 20 days pi from an independent donor, we have identified IgG positive cells, with a PC–like morphology, in cells with non-functional EBNA3A and EBNA3C (S10A Fig). These were observed approximately 3 times more frequently in cells with inactive EBNA3A/EBNA3C compared to EBV-infected cells expressing functional EBNA3A/EBNA3C (S10B Fig).

Taken together, these results show that in the absence of EBNA3A and EBNA3C, EBV-infected cells express an increasingly PC–like phenotype, with evidence of elevated IgG and IgM production, further indicating that EBNA3A and EBNA3C function to prevent PC differentiation.

## Discussion

We have provided an unambiguous link between the EBV transcription factors EBNA3A and EBNA3C, and the suppression of a transcription program that normally drives activated B cells towards the phenotype of terminally differentiated PC. Deletion or biochemical inactivation by manipulation of the *EBNA3A* and *EBNA3C* genes in the virus genome produces recombinant EBVs that, when used to infect CD19^+ve^ B cells ex vivo, are unable to produce ‘immortalized/transformed’ LCLs. Such viruses appear to have no gross defects that become manifest during the early phase of rapid B cell polyclonal expansion described by Luftig and colleagues ([20,53]). However, around 15 days pi, in cells where both EBNA3A and EBNA3C were functionally incapacitated, we saw substantially elevated levels of the CDKI p18^INK4c^ and the major PC differentiation transcriptional regulator BLIMP-1—unlike cells expressing functional EBNA3A and/or EBNA3C. Our results suggest that for maximal repression there is cooperation between EBNA3A and EBNA3C, but that each alone might exert repressor activity, in a donor dependent manner. This is consistent with reports of cooperative activity that have been described for other EBNA3A/EBNA3C-regulated genes [10,12,14,16,38,54]. The observation that these EBNA proteins can also be co-precipitated from cell extracts and the multiple reports that EBNA3A and EBNA3C commonly co-localize across the genome of LCLs (Figs 7 and 8; [10,14–16,41,55]) support this hypothesis. However, a precise biochemical description of the mechanism(s) responsible for cooperation has yet to be determined.

As both p18^INK4c^ and BLIMP-1 induce a post-mitotic state, it is not surprising that they have both been identified as tumour suppressor proteins that are commonly compromised by mutation or silencing in B cell lymphoma—including EBV-negative DLBCL [56–59]. In EBV-positive DLBCL it is possible that EBNA3A and EBNA3C are responsible for this inactivation and, in this setting, contribute to oncogenic progression.

We cannot rule out the possibility that EBNA3A and EBNA3C directly regulate additional genes required for differentiation. However, finding (by ChIP-seq and ChIP-qPCR) that both proteins are bound to chromatin at regulatory elements in the vicinity of the *CDKN2C* and *PRDM1* loci has driven our working hypothesis that these cellular regulators are primary targets and that EBNA3A/EBNA3C-binding to local chromatin results in the recruitment of factors that initiate and sustain the chromatin state responsible for reducing transcription. These data, and those described in many other reports, are consistent with EBNA3A and EBNA3C binding to regulatory elements, followed by the removal of activation-associated histone marks (e.g., H3/H4KAc), concomitant chromosomal conformational changes, and finally further covalent histone modifications by polycomb protein complexes (particularly H3K27me3, catalysed by the EZH2 component of PRC2). Polycomb-mediated modifications then fix this state into a stable epigenetic form of repression [12,18,60]. This sequence of events, with EBNA3A and EBNA3C initiating polycomb-mediated repression by 12 days pi—but not being required to sustain the state—could explain why repression of p18^INK4c^ and BLIMP-1 was not reversed by the inactivation of EBNA3A and EBNA3C, but can be reversed by the inhibitor GSK126 reducing global H3K27me3 (compare Figs 5, 6 and 9). Once the PRC2 machinery is in place, active EBNA3A and EBNA3C appear to be unnecessary at these specific loci to silence transcription in the infected cells and their progeny. Although it is beyond the scope of this study, it will be of great fundamental interest to determine what specific feature(s) of the *CDKN2C* (p18^INK4c^) and *PRDM1* (BLIMP-1) loci determine why they remain repressed after ablation of EBNA3A and EBNA3C, while simultaneously other EBNA3A/3C targets—including *BCL2L11* (BIM) and *CDKN2A* (p16^INK4a^)—are reactivated. Additionally, it will be of interest to determine whether this reversibility is related to requirements of gene expression during the sustained infection.

We believe that the biological aim of EBNA3A/EBNA3C-mediated epigenetic repression of p18^INK4c^ and BLIMP-1 is to suppress PC differentiation following EBV infection. This was confirmed through experiments showing cells with inactive EBNA3A and EBNA3C progress along a PC differentiation–like pathway following infection with EBV. We determined that physiological levels of both p18^INK4c^ and BLIMP-1 in EBNA3A/EBNA3C-null cells are sufficient to drive PC differentiation with expression comparable to, or exceeding, those in 2 PC models: U266 cells and in vitro induced PCs. With differentiation, associated BLIMP-1 target genes responded as predicted to elevated BLIMP-1 transcription levels. Furthermore, phenotypic consequences of increasing levels of differentiation factors were identified by FCA, with elevated levels of markers characteristic of PCs and increased IgG and IgM production in cells with inactive EBNA3A and EBNA3C at 20 days pi.

It is likely complete differentiation of cells with inactive EBNA3A and EBNA3C into terminally differentiated PCs is being prevented by additional consequences of EBV infection, alongside the consequences of inactive EBNA3A and EBNA3C. This likely includes reduced proliferative capacity and rising levels of the CDKI p16^INK4a^ leading to cell cycle arrest (S1 Fig, [39]). It would therefore be interesting to establish if p16^INK4a^-null cells with non-functional EBNA3A and EBNA3C can terminally differentiate into PCs.

**Fig 13:**
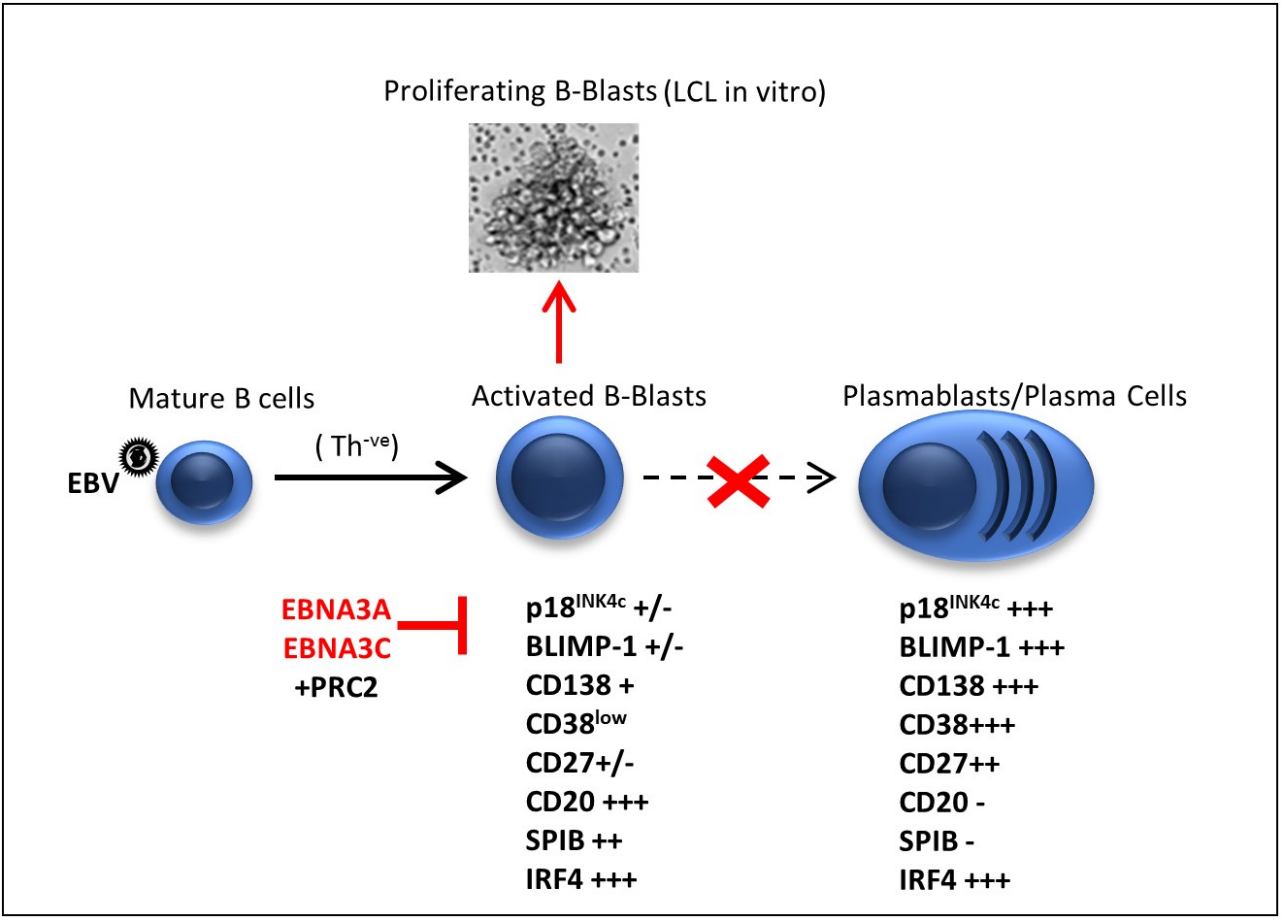
EBNA3A and EBNA3C inhibit the differentiation of Epstein-Barr virus (EBV)-activated B cells to plasma cells. Upon EBV infection, mature human B cells become activated, grow and proliferate [20]. In vivo, in the presence of T cells or T cell–derived factors, infected cells can enter the germinal centre and differentiate into memory B cells—the site of long-term EBV latency and persistence [6]. However, it has not been established what happens if T cell help is unavailable (Th^-ve^). Usually in the absence of T cell help, antigen-activated B cells can enter the default plasma cell differentiation pathway, resulting in antibody-producing plasma cells [28]. We suggest EBV has evolved to prevent default plasma cell differentiation, thus favouring latency in memory B cells, through specific repression of the plasma cell differentiation factors p18^INK4c^ and B lymphocyte-induced maturation protein-1 (BLIMP-1), by the viral transcription factors EBNA3A and EBNA3C that act in vitro to support the activated B-blast population in establishing continuously proliferating lymphoblastoid cell lines (LCLs). Since the repression of the p18^INK4c^ and BLIMP-1 genes utilizes the polycomb system (PRC2) and is stable and heritable, continuous expression of functional EBNA3A and EBNA3C is unnecessary to favour memory cell rather than plasma cell differentiation.

To our knowledge, p18^INK4c^ and BLIMP-1 are the first genes to be identified as epigenetically repressed by EBNA3 proteins, where sustained repression subsequently becomes independent of the EBNA3 proteins. This stable and heritable regulation involving PRC2 does not require continuous expression of EBNA3A and EBNA3C, which we believe relates to establishment of EBV latency in MBC [5]. When EBV infects any type of quiescent, mature human B cell, the consensus of opinion is that it becomes activated, grows, and within a few days begins to proliferate rapidly [20]. In vivo, if helper T cells or T cell–derived factors are available, these cells can differentiate into GC-like B cells, and as they progress through the GC, viral gene expression is sequentially silenced. Cells exit the GC as MBC which do not express viral proteins, and form the site of viral latency and persistence [6]. Usually, in the absence of EBV infection, when antigen-activated B cells have no T cell help, B cells can enter a default differentiation pathway to become plasmablasts, then Ig-secreting PCs [28]; however, it has yet to be established what happens if T cell help is unavailable in the context of EBV infection. We propose EBV has evolved a coordinated mechanism to favour long-term latency in MBC by preventing this PC differentiation pathway, whereby the transcription factors EBNA3A and EBNA3C specifically restrain the transcription of 2 critical regulators of PC differentiation—p18^INK4c^ and BLIMP-1. It is likely EBV has evolved to ensure transient expression of EBNA3A and EBNA3C during early EBV infection that is sufficient to irreversibly repress these PC differentiation factors as a mechanism to avoid of spontaneous PC differentiation of EBV-infected cells as EBNA3 genes are silenced during progression through the GC. This therefore favours memory B cell differentiation and long-term viral persistence and latency (summarized in Fig 13).

Finally, although the in vitro model system described here should be ideal for investigating how the switch from latency to the EBV lytic cycle and PC differentiation are coupled, we have been unable to shed light on this unifying feature of EBV biology. EBV replication occurs in rare cells undergoing PC differentiation in the tonsils of healthy carriers [9] and recently BLIMP-1 was demonstrated to induce EBV reactivation in B cells by activating transcription from the lytic switch promoters, R and Z [61], providing a molecular link between the 2 phenomena; however, this was found to occur in only a subset of B cells. A prediction, based on our experiments described above, was that in the conditional EBNA3A/EBNA3C model, expression of the EBV lytic-switch protein BZLF1 (and mRNA) would echo p18^INK4c^ and BLIMP-1 activation, with an inverse correlation between EBNA3A/EBNA3C function and EBV lytic activity. However, we found no consistent correlation between inactivating EBNA3A and EBNA3C, p18^INK4c^ and BLIMP-1 activation, and BZLF1 mRNA or protein expression. Donor-dependent behaviour, or incomplete terminal differentiation of EBNA3A/EBNA3C-null cells, may be responsible for this. On the other hand, it may result from the rarity in which EBV reactivation occurs [9], of which a contributing factor may be difficulty in overcoming the EBNA3A and EBNA3C mediated stable repression of p18^INK4c^ and BLIMP-1. Unfortunately, exploring the biochemistry linking activation and differentiation is very challenging and beyond the scope of this current study.

In summary, we have formally established a link between the transcription regulatory activities of EBV and the B cell-to-PC differentiation process, and provided unique insights into the molecular mechanisms involved. We have shown that through epigenetic repression of the differentiation factors p18^INK4c^ and BLIMP-1, EBNA3A and EBNA3C suppress the PC differentiation pathway following EBV infection, thus favouring establishment of long-term EBV latency in MBC.

## Materials and methods

### Ethics statement

The buffy coat residues used in this study for the isolation of CD19^+ve^ primary B cells were purchased from the UK Blood Transfusion Service. As these were derived from anonymous volunteer blood donors, no ethical approval is required.

### Construction of recombinant EBV-BAC EBNA3C-ERT2 and EBNA3A-ERT2/3C-ERT2

The HT-sensitive oestrogen receptor ERT2 used to construct EBNA3A-ERT2 [10] was used to construct EBNA3C-ERT2 fusion proteins in the B95-8 EBV background. These fusions were recombined into a B95-8 BAC lacking EBNA3C or all the EBNA3s, respectively, using methods previously described [10,38,62] to produce BACs containing either EBNA3C-ERT2 (EBNA3C-ERT2) independently or both EBNA3A-ERT2 and EBNA3C-ERT2 (EBNA3A/3C-ERT2). Restriction digest and pulse field gel electrophoresis were used to confirm BAC structures.

### Cell culture

Cells were routinely cultured at 10% CO_2_ and 37°C in RPMI 1640 medium supplemented 10% foetal calf serum (FCS), penicillin, and streptomycin. Cells were routinely seeded at 3 x 10^5^/ml 1 day before harvesting. The activating ligand HT was added to 400 nM and EZH2 inhibitor (GSK126) to 4 μM, where indicated. Both these supplements were added to cultures every time fresh medium was added to the cells. Where indicated, medium from cultures containing HT was exchanged for fresh media noted as ‘wash’ cell populations. U266 multiple myeloma cells were a kind gift from Professor Anastasios Karadimitris (Imperial College London, UK).

### B cell infection and time courses

Recombinant viruses EBNA3KO, ‘wild type’ (WT, B95-8-BAC) [11], EBNA3A-ERT2 [10], EBNA3C-ERT (this study), and EBNA3A/EBNA3C-ERT2 (this study), were produced and titred as described previously [38]. Primary B cells were isolated from PBLs obtained from anonymous buffy coat donors (UK Blood Transfusion Service) by centrifugation over Ficoll. CD19 microbeads were used for magnetic separation of purified B cells using an autoMACS separator (Miltenyi Biotec). Virus amounts were normalised across infections by Raji green units (RGU) [38] and cells were cultured initially in 15% FCS, reduced to 10% after 30 days.

### qPCR

RNA was isolated from cells using an RNeasy mini kit (Qiagen) with DNAse digestion as per the manufacturer’s instructions. Reverse transcription of RNA into cDNA was performed using Superscript III First Strand Synthesis Supermix for qRT-PCR (Invitrogen). Ten nanograms of cDNA was run per qPCR reaction using Platinum SYBR Green qPCR Supermix UDG kit (Invitrogen), and performed on an ABI 7900HT real-time PCR machine. The comparative Ct (ΔΔCt) method was used to calculate relative mRNA expression with the housekeeping gene *GNB2L1* used as an endogenous control. Gene expression is relative to either uninfected primary B cells or LCLs grown with HT as indicated. Primer sequences for this study are listed in S1 Table. Error bars in figures are the standard deviation from 3 triplicate qPCR replicates for each mRNA sample.

### Immunoblotting

SDS polyacrylamide gel electrophoresis and western blotting was performed as described previously [10, 17, 39]. Antibodies used in this study are listed in S2 Table.

### ChIP

ChIP assay and qPCR analysis were performed as described previously [12]. Antibodies and sequences of primers used in these assays are listed in S2 Table and S3 Table, respectively.

### FCA

For FCA, cells were harvested, washed once in PBS, and then incubated with 1 ml PBS with 0.5 μl Fixable Viability Stain 780 for 10 minutes at room temperature. Cells were then washed once in PBS, once in PBS 1% BSA, and then stained for cell surface markers in PBS 1% BSA for 30 minutes at 4°C by resuspending in 100 μl 1% PBS/BSA containing the conjugated antibodies anti-CD20-AF700 (5 μl), anti-CD27-BV605 (1 μl), anti-CD38-PE-Cy7 (2.5 μl), and anti-CD138-APC (5 μl). For Ig analysis, following viability staining (as above) cells were additionally fixed using a Cytofix/Cytoperm Fixation/Permeabilization Kit (BD Biosciences) as per the manufacturer’s instructions. Cells were then stained for intracellular Ig in PBS 1% BSA for 30 minutes at 4°C by resuspending in 100 μl PBS 1% BSA containing the conjugated antibodies anti-IgG-PE and IgM-BV605. Following staining, cells were washed twice in PBS and fluorescence was measured on a Fortessa B flow cytometer. Gating was used to exclude dead cells and doublets, and analysis and mean fluorescence intensity calculation was performed using FlowJo software. All antibodies were purchased from BD Biosciences. Representative ancestry gating strategies and FCS files can be found at osf.io/97zrj.

### In vitro PC induction

PCs were induced essentially as described previously [51]. Briefly, CD19^+ve^ primary B cells were purified, and CD40-ligand (Enzo Life Sciences) and IL21 (Preprotech) were added to cell culture at a final concentration of 50 ng/ml and 100 ng/ml, respectively. Cells were harvested at 7 days post induction.

### Immunofluorescence

Cytospins of 1 x 10^5^ cells were fixed in methanol/acetone, rehydrated in PBS, and stained for IgG as described previously [26]. IgG positive cells were counted [(HT+ 6/210, 14/391), (HT- 22/206, 43/500)].

### Cell cycle analysis

Cell proliferation assays were performed as described previously [39]. Briefly, cells were harvested after 2 hours in the presence of 5 μM of EdU and stained with Live/Dead Fixable Violet Cell Stain. Following ethanol fixation and rehydration, click chemistry was used for EdU labelling before staining with FxCycle Far Red to stain for DNA content. Fluorescence was measured on an LSR II flow cytometer; dead cells and doublets were excluded from analysis.

## Supporting information

S1 Fig EBNA3A and EBNA3C repress *CDKN2A* (p16^INK4a^) transcription but not *ALAS1*. CD19^+ve^ purified B cells were infected with 3AERT2 (**A** and **D**), 3CERT2 (**B** and **E**), and 3A3CERT2 (**C** and **F**) recombinant EBV and cultured for 30 days with (+) or without (-) HT. RNA samples were taken at the times after infection indicated and qPCR analysis performed. *ALAS1* (control housekeeping gene, **A**, **B**, **C**) and *CDNK2C* (p16^INK4a^; **D**, **E**, **F**) relative mRNA expression was normalised to the endogenous control *GNB2L1* and fold change is shown relative to uninfected B cells at day 0. Error bars show the standard deviation of qPCR triplicates for each sample. Analysis of HT (-) infected cells at later time points was not always possible because of large amounts of cell death in the culture. Numerical data for this figure can be found at osf.io/97zrj.
(TIFF)

S2 Fig EBNA3A and EBNA3C repress expression of p15^INK4b^. CD19^+ve^ purified B cells from 3 donors (D1, D2, D3) were infected with 3A3CERT2 recombinant EBV and cultured with (+) or without (-) HT for 20 days (**A**), and established conditional LCLs from 3 donors (D1, D2, D3) were cultured with (+) or washed and grown without (WASH) HT for 30 days (**B**). Analysis of expression of *CDKN2B* (p15^INK4b^) mRNA was performed by qPCR and relative mRNA expression was normalised to the endogenous control *GNB2L1*, with mean fold change shown relative to cells grown with (+) HT. Error bars indicate the standard deviation of qPCR triplicates for each sample. Numerical data for this figure can be found at osf.io/97zrj.
(TIFF)

S3 Fig Transcription of p18^INK4c^ and BLIMP-1 is repressed early after infection with EBV. CD19^+ve^ purified B cells from one independent donor were infected with 3A3CERT2 recombinant EBV and cultured for 30 days with HT (+), without HT (-), or HT was removed after 12 or 4 days (WASH), as indicated. RNA samples were taken at the times after infection indicated and qPCR analysis performed. *CDKN2C* (p18^INK4c^; **A** and **D**), *PRDM1* (BLIMP-1; **B** and **E**), and *ALAS1* (control housekeeping gene, **C** and **F**) relative mRNA expression was normalised to the endogenous control *GNB2L1* with fold change shown relative to uninfected B cells at day 0. Error bars show the standard deviation of qPCR triplicates for each sample. Analysis of HT (-) and day 4 washed infected cells at later time points was not possible because of large amounts of cell death in the culture. Numerical data for this figure can be found at osf.io/97zrj.
(TIFF)

S4 Fig Treatment of 3A3CERT2-infected cells with the EZH2 inhibitor GSK126. Established ‘WT’ (B98.5-BAC) LCLs from 2 different donors (LCL WT D1 and LCL WT D2) were treated with the EZH2 inhibitor GSK126 for 20 days. (**A**) Western blotting extracts of the cells show expression of EBNA3A and EBNA3C; γ-tubulin was used as a loading control; molecular weight markers are shown in kDa. (**B**) Cell cycle distribution of treated cells was assessed by EdU incorporation (5 μM) over 2 hours and determined by flow cytometry. Number of cells at each stage of the cell cycle is shown as a percentage of live single cells.
(TIFF)

S5 Fig Regulation of well-characterized BLIMP-1 target genes in EBNA3A/EBNA3C-null cells. CD19^+ve^ purified B cells were infected with 3A3CERT2 recombinant EBV and cultured with (+) or without (-) HT (**A**,**C,E,G**) for 30 days, or with EBNA3KO and ‘WT’ (B95.8-BAC) (**B**,**D**,**F**,**H**) and cultured for 30 days. RNA samples were taken at the times after infection indicated and qPCR analysis performed. *PRDM1* (BLIMP-1, **A** and **B**), *SPIB* (**C** and **D**), *LMO2* (**E** and **F**), and *XBP1* (**G** and **H**) relative mRNA expression was normalised to the endogenous control *GNB2L1* and fold change is shown relative to uninfected B cells at day 0. Error bars show the standard deviation of qPCR triplicates for each sample. Analysis of HT (-) infected cells at later time points was not possible because of large amounts of cell death in the culture. Numerical data for this figure can be found at osf.io/97zrj.
(TIFF)

S6 Fig IRF4 protein levels are unaffected by EBNA3A/EBNA3C function. Expression of IRF4 shown by western blotting extracts from 3A3CERT2-infected CD19^+ve^ primary B cells from 2 donors (1°B D1, 1°B D2) grown with (+) or without (-) HT for 20 days and 3A3CERT2 conditional LCLs from 2 donors (LCL D1, LCL D2) grown with HT (+) or washed and grown without HT for 30 days (W). An extract from the myeloma/plasmacytoma cell line U266 is shown for comparison. In each blot, γ-tubulin was used as a loading control, and molecular weight markers are shown in kDa.
(TIFF)

S7 Fig Flow cytometric profiles of EBNA3A/EBNA3C-null cells are consistent with the plasmablast/plasma cell phenotype. U266 cells (black) and CD19^+ve^ purified B cells were infected with 3A3CERT2 recombinant EBV and cultured with (+, blue) or without (-, red) HT for 20 days, then analysed for CD138, CD38, CD27, and CD20 expression by flow cytometry. (**A**) Contour plots show CD38 and CD138 surface expression; the quadrant value represents the percentage of live single cells expressing both markers. Histograms show expression of CD138 (**B**), CD38 (**C**), CD27 (**D**), and CD20 (**E**). Results shown are one representative example from at least two independent experiments.
(TIFF)

S8 Fig Expression of p18^INK4c^ and BLIMP-1 mRNA in 3A3CERT2-infected cells and CD40-L/IL21 induced plasma cells. RNA samples were taken from CD19^+ve^ purified B cells from 2 donors at day 0 (d0) and 7 days post induction (d7) with CD40-L/IL21 (PC1, PC2), and CD19^+ve^ purified B cells from 3 independent donors (D1, D2, D3) infected with 3A3CERT2 recombinant EBV and grown for 20 days with (+) or without (-) HT, and quantified by qPCR. (**A**) *CDKN2C* (p18^INK4c^) and (**B**) *PRDM1* (BLIMP-1) relative mRNA expression was normalised to the endogenous control *GNB2L1* with fold change shown relative to D1 grown with HT (+). Error bars show the standard deviation of qPCR triplicates for each sample. Numerical data for this figure can be found at osf.io/97zrj.
(TIFF)

S9 Fig Recombinant EBV-infected cells express IgG and IgM. CD19^+ve^ purified B cells were infected with 3A3CERT2 recombinant EBV and cultured with (+, blue) or without (-, red) HT for 20 days, then analysed for IgG and IgM expression by flow cytometry. Histograms show expression of IgG (**A**) and IgM (**B**). CD40-ligand/IL21 induced cells were used as a control (orange). Histograms shown are one representative example from at least two independent experiments.
(TIFF)

S10 Fig IgG expression is elevated when EBNA3A and EBNA3C are inactive. Cytospins of CD19^+ve^ purified B cells infected with 3A3CERT2 recombinant EBV and cultured with (+) or without (-) HT for 20 days were stained with a FITC-conjugated Rabbit anti-human IgG antibody and examined by fluorescence microscopy. The levels of cytoplasmic IgG found in the LCL -/+HT were compared. Cells showing high levels of cytoplasmic IgG are indicated by arrows (**A**); percentage of IgG positive cells is also shown (**B**). Numerical data for this figure can be found at osf.io/97zrj.
(TIFF)

S1 Table List of gene expression qRT-PCR primers sequences used in the study. (TIFF)

S2 Table List of antibodies used in the study. (TIFF)

S3 Table List of ChIP-qPCR primer sequences used in the study. (TIFF)

## Abbreviations

BAC: bacterial artificial chromosome
BL: Burkitt lymphoma
BLIMP-1: B lymphocyte-induced maturation protein-1
CDKI: cyclin-dependent kinase inhibitor
ChIP-qPCR: chromatin immunoprecipitation quantitative PCR
ChIP-seq: chromatin immunoprecipitation sequencing
DLBCL: diffuse large B cell lymphoma
EBV: Epstein-Barr virus
EZH2: Enhancer of Zeste Homologue-2
FCA: flow cytometric analysis
GC: germinal centres
HT: 4-hydroxytamoxifen
Ig: immunoglobulin
IRF4: interferon-regulatory factor 4
LCL: lymphoblastoid cell line
MACS: model-based analysis of ChIP-seq
MBC: memory B cells
NK: natural killer cells
PC: plasma cell
pi: post-infection
PRC2: polycomb repressor complex 2
PTLD: post-transplant lymphoproliferative disease
qPCR: quantitative PCR
Th: T helper lymphocytes
TSS: transcription start site
XBP-1: X-box binding protein-1
