# Supplementary figures and images for: EBV epigenetically suppresses the B cell-to-plasma cell differentiation pathway while establishing long-term latency
Source: PLoS Biol. 2017 Aug 3;15(8):e2001992. doi: 10.1371/journal.pbio.2001992 (PMC5542390; doi:10.1371/journal.pbio.2001992)

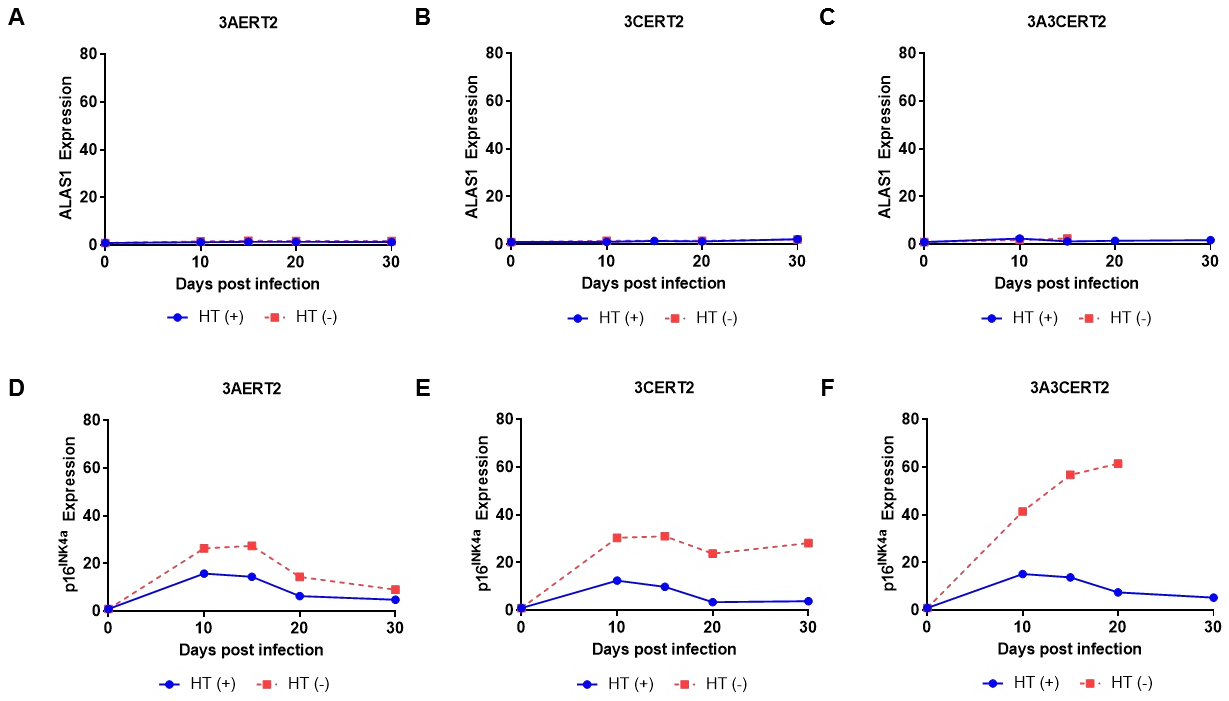

Supplement: S1 Fig — CD19+ve purified B cells were infected with 3AERT2 (A and D), 3CERT2 (B and E), and 3A3CERT2 (C and F) recombinant EBV and cultured for 30 days with (+) or without (-) HT. RNA samples were taken at the times after infection indicated and qPCR analysis performed. ALAS1 (control housekeeping gene, A, B, C) and CDNK2C (p16INK4a; D, E, F) relative mRNA expression was normalised to the endogenous control GNB2L1 and fold change is shown relative to uninfected B cells at day 0. Error bars show the standard deviation of qPCR triplicates for each sample. Analysis of HT (-) infected cells at later time points was not always possible because of large amounts of cell death in the culture. Numerical data for this figure can be found at osf.io/97zrj. (TIFF) [file pbio.2001992.s001.TIFF]

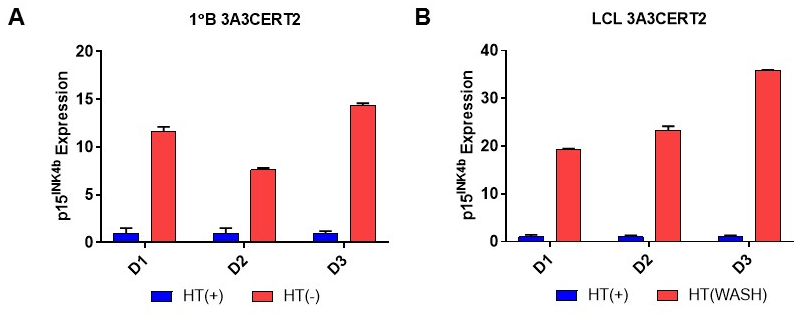

Supplement: S2 Fig — CD19+ve purified B cells from 3 donors (D1, D2, D3) were infected with 3A3CERT2 recombinant EBV and cultured with (+) or without (-) HT for 20 days (A), and established conditional LCLs from 3 donors (D1, D2, D3) were cultured with (+) or washed and grown without (WASH) HT for 30 days (B). Analysis of expression of CDKN2B (p15INK4b) mRNA was performed by qPCR and relative mRNA expression was normalised to the endogenous control GNB2L1, with mean fold change shown relative to cells grown with (+) HT. Error bars indicate the standard deviation of qPCR triplicates for each sample. Numerical data for this figure can be found at osf.io/97zrj. (TIFF) [file pbio.2001992.s002.TIFF]

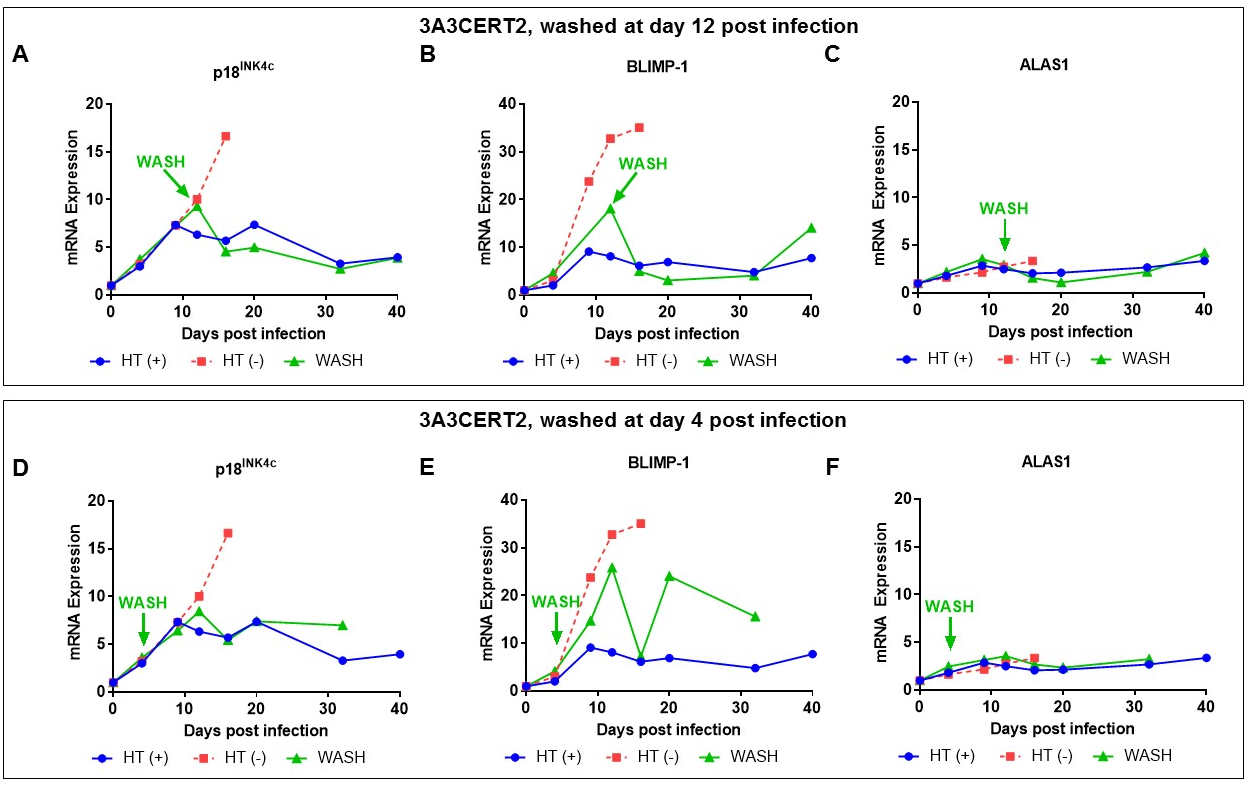

Supplement: S3 Fig — CD19+ve purified B cells from one independent donor were infected with 3A3CERT2 recombinant EBV and cultured for 30 days with HT (+), without HT (-), or HT was removed after 12 or 4 days (WASH), as indicated. RNA samples were taken at the times after infection indicated and qPCR analysis performed. CDKN2C (p18INK4c; A and D), PRDM1 (BLIMP-1; B and E), and ALAS1 (control housekeeping gene, C and F) relative mRNA expression was normalised to the endogenous control GNB2L1 with fold change shown relative to uninfected B cells at day 0. Error bars show the standard deviation of qPCR triplicates for each sample. Analysis of HT (-) and day 4 washed infected cells at later time points was not possible because of large amounts of cell death in the culture. Numerical data for this figure can be found at osf.io/97zrj. (TIFF) [file pbio.2001992.s003.TIFF]

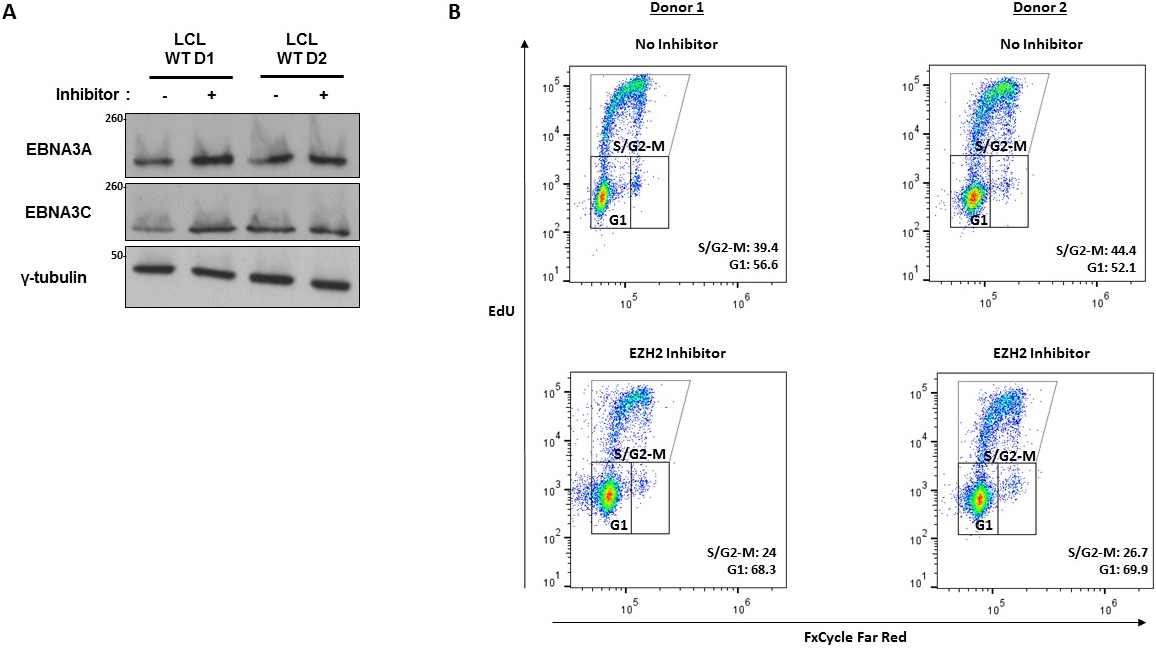

Supplement: S4 Fig — Established ‘WT’ (B98.5-BAC) LCLs from 2 different donors (LCL WT D1 and LCL WT D2) were treated with the EZH2 inhibitor GSK126 for 20 days. (A) Western blotting extracts of the cells show expression of EBNA3A and EBNA3C; γ-tubulin was used as a loading control; molecular weight markers are shown in kDa. (B) Cell cycle distribution of treated cells was assessed by EdU incorporation (5 μM) over 2 hours and determined by flow cytometry. Number of cells at each stage of the cell cycle is shown as a percentage of live single cells. (TIFF) [file pbio.2001992.s004.TIFF]

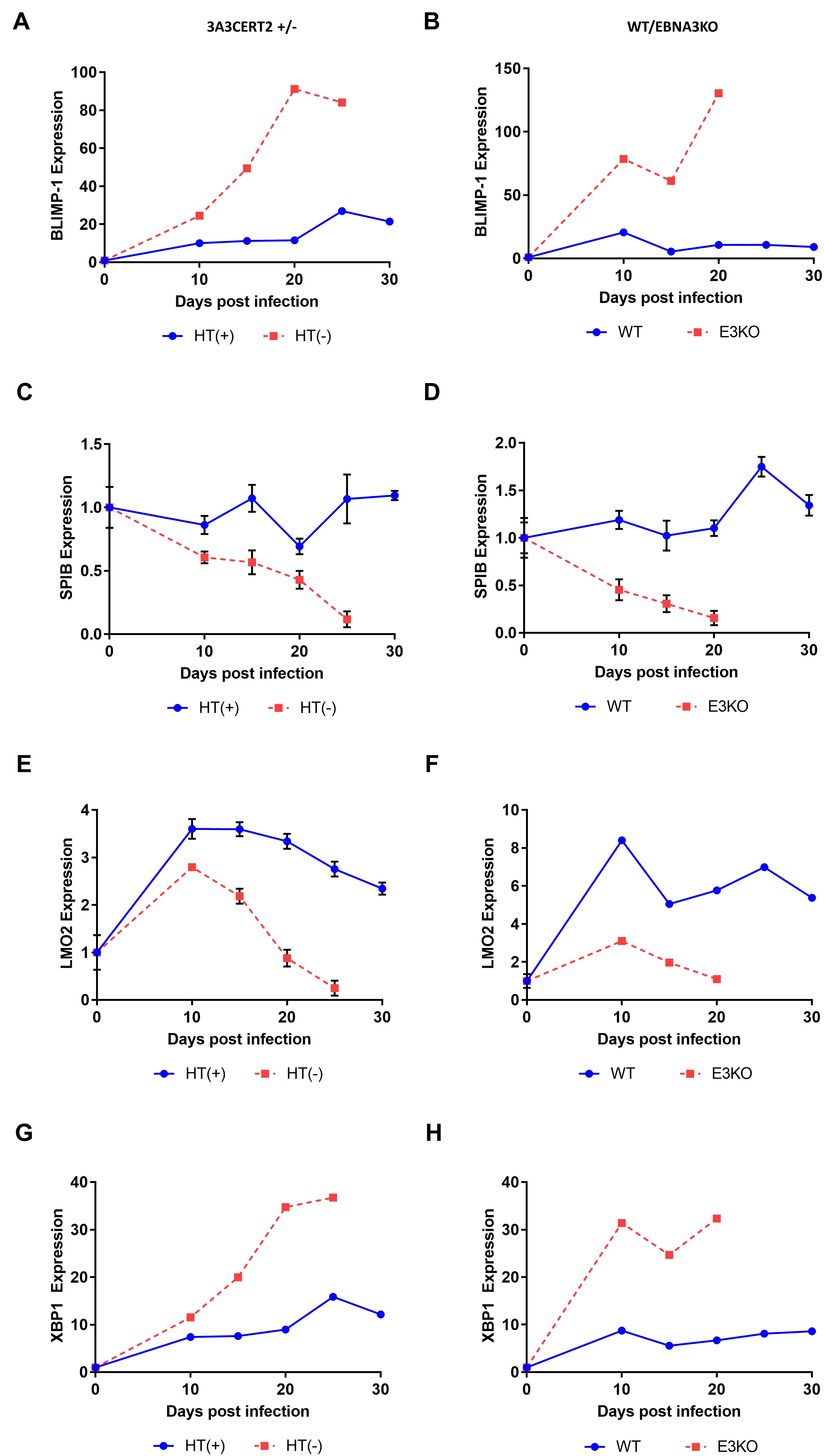

Supplement: S5 Fig — CD19+ve purified B cells were infected with 3A3CERT2 recombinant EBV and cultured with (+) or without (-) HT (A,C,E,G) for 30 days, or with EBNA3KO and ‘WT’ (B95.8-BAC) (B,D,F,H) and cultured for 30 days. RNA samples were taken at the times after infection indicated and qPCR analysis performed. PRDM1 (BLIMP-1, A and B), SPIB (C and D), LMO2 (E and F), and XBP1 (G and H) relative mRNA expression was normalised to the endogenous control GNB2L1 and fold change is shown relative to uninfected B cells at day 0. Error bars show the standard deviation of qPCR triplicates for each sample. Analysis of HT (-) infected cells at later time points was not possible because of large amounts of cell death in the culture. Numerical data for this figure can be found at osf.io/97zrj. (TIFF) [file pbio.2001992.s005.TIFF]

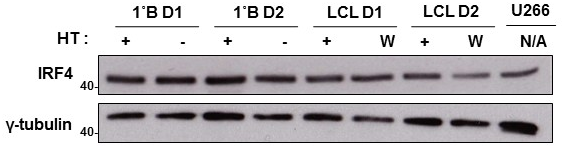

Supplement: S6 Fig — Expression of IRF4 shown by western blotting extracts from 3A3CERT2-infected CD19+ve primary B cells from 2 donors (1°B D1, 1°B D2) grown with (+) or without (-) HT for 20 days and 3A3CERT2 conditional LCLs from 2 donors (LCL D1, LCL D2) grown with HT (+) or washed and grown without HT for 30 days (W). An extract from the myeloma/plasmacytoma cell line U266 is shown for comparison. In each blot, γ-tubulin was used as a loading control, and molecular weight markers are shown in kDa. (TIFF) [file pbio.2001992.s006.TIFF]

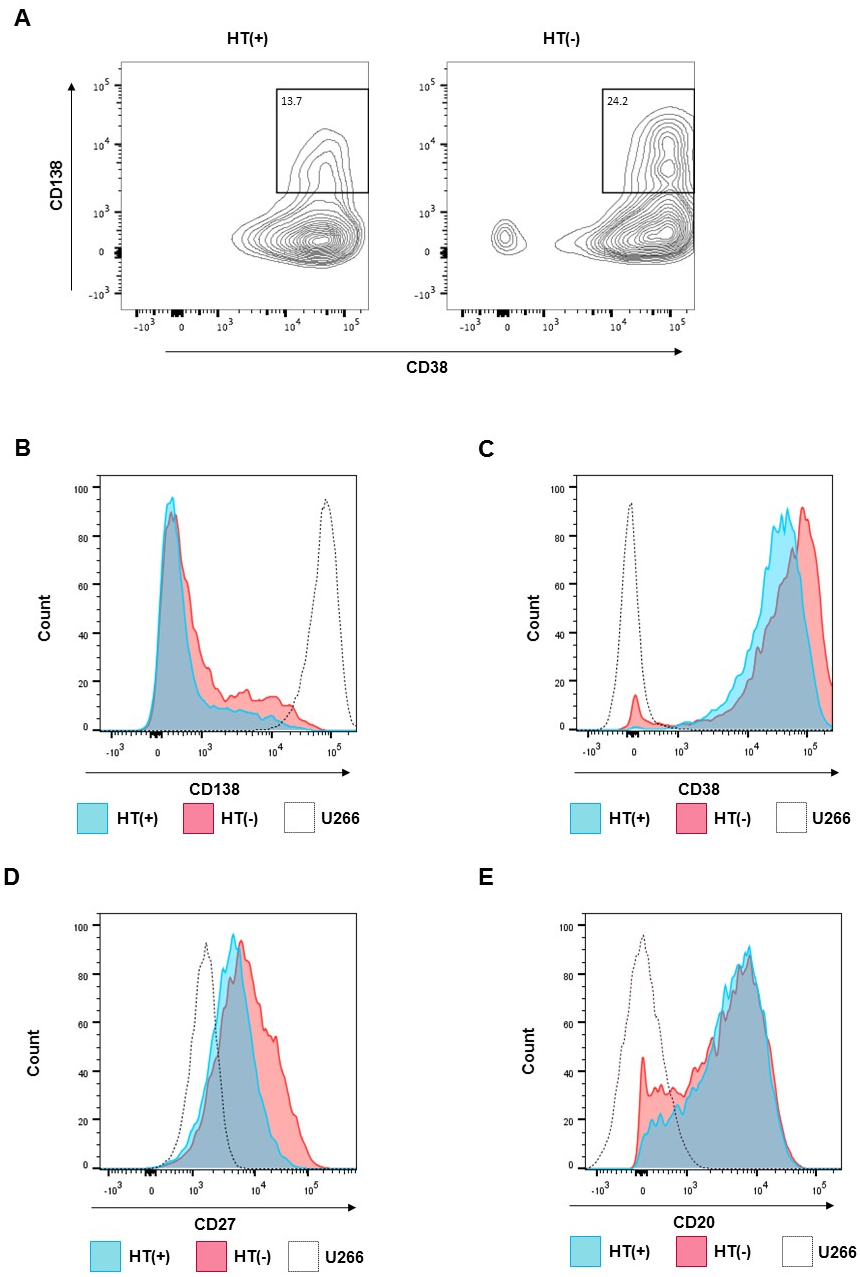

Supplement: S7 Fig — U266 cells (black) and CD19+ve purified B cells were infected with 3A3CERT2 recombinant EBV and cultured with (+, blue) or without (-, red) HT for 20 days, then analysed for CD138, CD38, CD27, and CD20 expression by flow cytometry. (A) Contour plots show CD38 and CD138 surface expression; the quadrant value represents the percentage of live single cells expressing both markers. Histograms show expression of CD138 (B), CD38 (C), CD27 (D), and CD20 (E). Results shown are one representative example from at least two independent experiments. (TIFF) [file pbio.2001992.s007.TIFF]

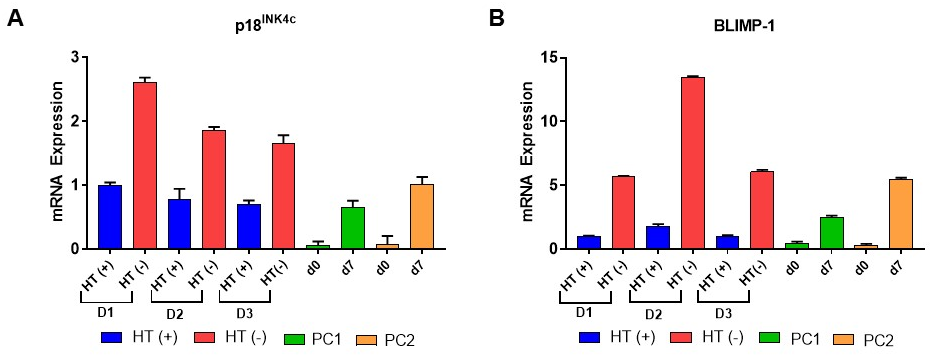

Supplement: S8 Fig — RNA samples were taken from CD19+ve purified B cells from 2 donors at day 0 (d0) and 7 days post induction (d7) with CD40-L/IL21 (PC1, PC2), and CD19+ve purified B cells from 3 independent donors (D1, D2, D3) infected with 3A3CERT2 recombinant EBV and grown for 20 days with (+) or without (-) HT, and quantified by qPCR. (A) CDKN2C (p18INK4c) and (B) PRDM1 (BLIMP-1) relative mRNA expression was normalised to the endogenous control GNB2L1 with fold change shown relative to D1 grown with HT (+). Error bars show the standard deviation of qPCR triplicates for each sample. Numerical data for this figure can be found at osf.io/97zrj. (TIFF) [file pbio.2001992.s008.TIFF]

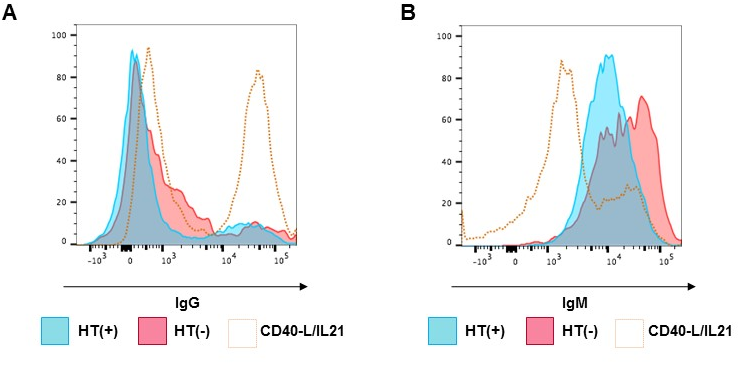

Supplement: S9 Fig — CD19+ve purified B cells were infected with 3A3CERT2 recombinant EBV and cultured with (+, blue) or without (-, red) HT for 20 days, then analysed for IgG and IgM expression by flow cytometry. Histograms show expression of IgG (A) and IgM (B). CD40-ligand/IL21 induced cells were used as a control (orange). Histograms shown are one representative example from at least two independent experiments. (TIFF) [file pbio.2001992.s009.TIFF]

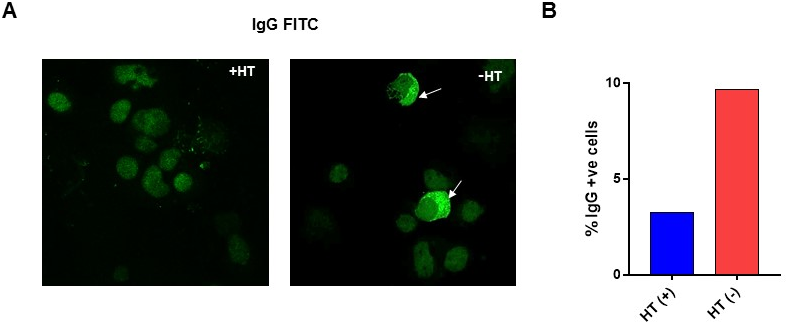

Supplement: S10 Fig — Cytospins of CD19+ve purified B cells infected with 3A3CERT2 recombinant EBV and cultured with (+) or without (-) HT for 20 days were stained with a FITC-conjugated Rabbit anti-human IgG antibody and examined by fluorescence microscopy. The levels of cytoplasmic IgG found in the LCL -/+HT were compared. Cells showing high levels of cytoplasmic IgG are indicated by arrows (A); percentage of IgG positive cells is also shown (B). Numerical data for this figure can be found at osf.io/97zrj. (TIFF) [file pbio.2001992.s010.TIFF]

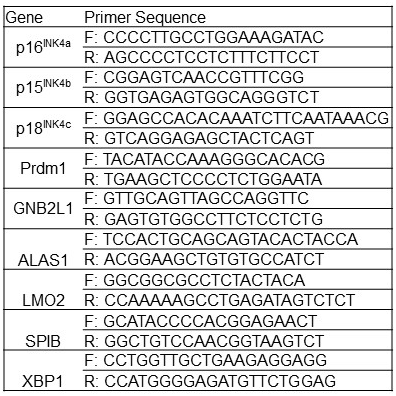

Supplement: S1 Table — (TIFF) [file pbio.2001992.s011.TIFF]

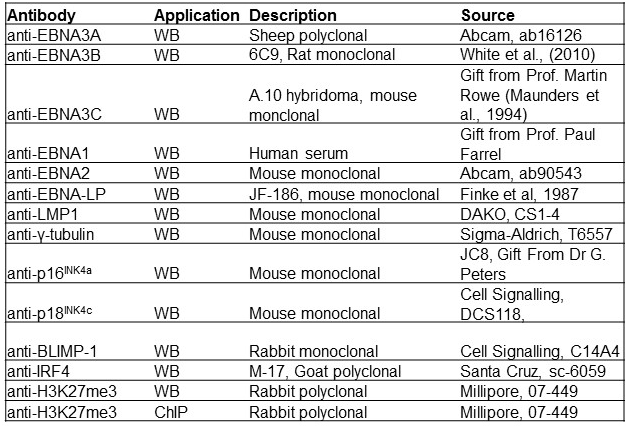

Supplement: S2 Table — (TIFF) [file pbio.2001992.s012.TIFF]

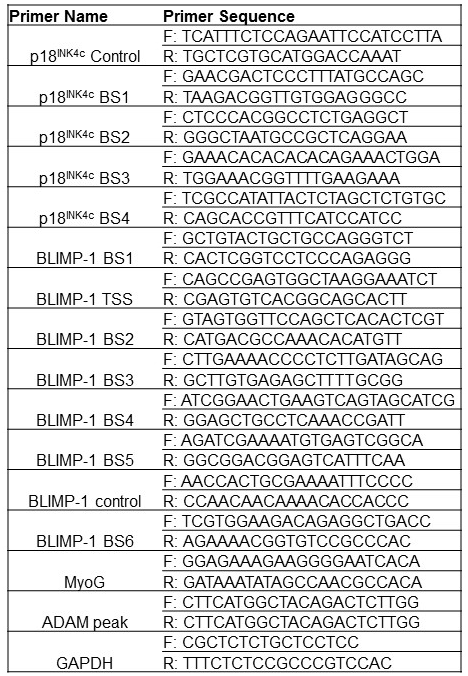

Supplement: S3 Table — (TIFF) [file pbio.2001992.s013.TIFF]
